# Carbon Adsorbent Properties Impact Hydrated Electron Activity and Perfluorocarboxylic Acid (PFCA) Destruction

**DOI:** 10.1021/acsestengg.4c00211

**Published:** 2024-08-02

**Authors:** Hosea A. Santiago-Cruz, Zimo Lou, Jiang Xu, Ryan C. Sullivan, Bailey B. Bowers, Rachel A. Molé, Wan Zhang, Jinghao Li, Joshua S. Yuan, Susie Y. Dai, Gregory V. Lowry

**Affiliations:** † Department of Civil and Environmental Engineering, Carnegie Mellon University, Pittsburgh, Pennsylvania 15213, United States; ‡ Collaborative Innovation Center of Yangtze River Delta Region Green Pharmaceuticals, Zhejiang University of Technology, Hangzhou 310014, China; § College of Environmental and Resource Sciences, Zhejiang University, Hangzhou 310058, China; ∥ Department of Chemistry, Carnegie Mellon University, Pittsburgh, Pennsylvania 15217, United States; ⊥ Department of Chemistry and Biochemistry, Oberlin College, Oberlin, Ohio 44074, United States; # Department of Plant Pathology and Microbiology, Texas A&M University, College Station, Texas 77843, United States; ∇ Department of Energy, Environmental, and Chemical Engineering, McKelvey School of Engineering, Washington University in St. Louis, St. Louis, Missouri 63130-4899, United States; ¶ Department of Mechanical Engineering, Carnegie Mellon University, Pittsburgh, Pennsylvania 15217, United States

**Keywords:** PFAS, hydrated electron, carbon adsorbents, regeneration, advanced reduction process

## Abstract

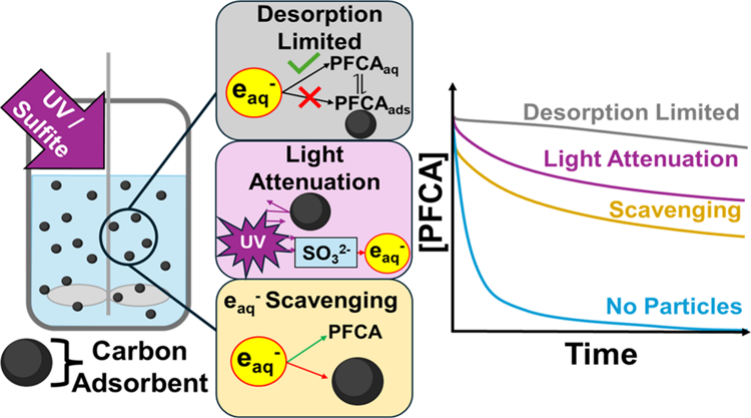

Carbon-based adsorbents used to remove recalcitrant water contaminants, including perfluoroalkyl substances (PFAS), are often regenerated using energy-intensive treatments that can form harmful byproducts. We explore mechanisms for sorbent regeneration using hydrated electrons (e_aq_^–^) from sulfite ultraviolet photolysis (UV/sulfite) in water. We studied the UV/sulfite treatment on three carbon-based sorbents with varying material properties: granular activated carbon (GAC), carbon nanotubes (CNTs), and polyethylenimine-modified lignin (lignin). Reaction rates and defluorination of dissolved and adsorbed model perfluorocarboxylic acids (PFCAs), perfluorooctanoic acid (PFOA) and perfluorobutanoic acid (PFBA), were measured. Monochloroacetic acid (MCAA) was employed to empirically quantify e_aq_^–^ formation rates in heterogeneous suspensions. Results show that dissolved PFCAs react rapidly compared to adsorbed ones. Carbon particles in solution decreased aqueous reaction rates by inducing light attenuation, e_aq_^–^ scavenging, and sulfite consumption. The magnitude of these effects depended on adsorbent properties and surface chemistry. GAC lowered PFOA destruction due to strong adsorption. CNT and lignin suspensions decreased e_aq_^–^ formation rates by attenuating light. Lignin showed high e_aq_^–^ quenching, likely due to its oxygenated functional groups. These results indicate that desorbing PFAS and separating the adsorbent before initiating PFAS degradation reactions will be the best engineering approach for adsorbent regeneration using UV/sulfite.

## Introduction

Perfluoroalkyl substances (PFAS) are anthropogenic fluorochemicals frequently detected in surface waters, treated drinking water, and groundwater.^1−3^ Because of their ubiquity,^4−6^ extreme persistence,^7,8^ and toxicity,^9−11^ several perfluoroalkyl acids (PFAAs) are regulated within the nanograms per liter (ng/L) range in drinking water.^12^ This highlights the significance of PFAA removal as a health priority and a strenuous engineering challenge. Carbon-based adsorbents like granular activated carbon (GAC) are frequently employed to remove PFAS from water because of their high sorption capacity and low cost, given that there are few other efficient alternatives to remove trace water pollutants.^13−15^ Once adsorbents are spent, they are disposed of in landfills or regenerated.^16,17^ GAC can be regenerated with several techniques such as electrochemical processes,^18^ but it is often regenerated at scale through thermal treatments,^16^ which can simultaneously decompose PFAS and achieve defluorination at higher temperatures (700–1000 °C).^17,19,20^ However, thermal processes are energy-intensive, costly, and could emit organofluorine byproducts formed from incomplete destruction.^5,16,20^ Therefore, there is a need to develop alternative adsorbent regeneration techniques that can simultaneously mineralize PFAS.

Advanced reduction processes (ARPs) provide an attractive, destructive scheme for PFAAs. Hydrated electrons (e_aq_^–^) produced by ultraviolet (UV) photolysis of source chemicals (e.g., sulfite, iodide, indole-derivatives) are highly reactive nucleophilic species (*E*^0^ = −2.9 V)^21^ that can degrade PFAAs in water with high defluorination efficiencies.^22−24^ Although ARPs can degrade PFAAs at relatively low concentrations (μg/L),^25^ the cost of implementing ARPs for concentrations of PFAAs typically found in water (ng/L to low μg/L) may be a limitation for treatment of large volumes of water without preconcentration of PFAS.^26,27^ Therefore, ARPs would only be practical after a concentration step.^26−28^

Combining the high reactivity of e_aq_^–^ with preconcentration using a carbon sorbent offers the opportunity to degrade adsorbed PFAA while regenerating spent sorbents simultaneously. Perfluorooctanesulfonic acid (PFOS) was successfully degraded via e_aq_^–^ when coadsorbed with the source chemical (indole-derivative) under UV irradiation on a montmorillonite surface due to the proximity of the PFOS and the hydrated electron source.^29^ The rate of PFOS defluorination increased as the surface loading of PFOS increased. In contrast, using sulfite (SO_3_^2–^) as the hydrated electron source, perfluorooctanoic acid (PFOA) adsorbed onto an ion-exchange resin was less reactive with e_aq_^–^ compared to dissolved PFOA, which rapidly decomposes.^30^ This suggests that the desorption rate of PFOA from the ion-exchange resin may limit its destruction rate. Moreover, UV/sulfite treatment of PFOA in a biochar suspension resulted in lower defluorination than the no particle case at high pH (pH 8–10).^31^ In the absence of clear trends or mechanistic interpretations of the effect of adsorption on PFAA reactivity with e_aq_^–^, there is a need to better understand the factors impacting e_aq_^–^ reactivity in heterogeneous systems.

Carbon sorbents may impact the e_aq_^–^ reactivity beyond the effects of PFAA solid–water partitioning. In UV-enabled ARPs, PFAA reaction rates are controlled by the generation rate of hydrated electrons (*R*_f_^e_aq_–^) and the competing reactions of scavengers, which consume available e_aq_^–^.^32^ The elemental reactions describing the kinetics of target contaminant degradation are as follows
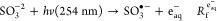
1
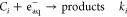
2
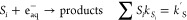
3

Equation 1 illustrates the UV photolysis of sulfite (SO_3_^–2^) to generate hydrated electrons, i.e., the e_aq_^–^ formation rate, *R*_f_^e_aq_–^ (M s^–1^). More detail on the definition of *R*_f_^e_aq_–^ is described in Text S1 and elsewhere.^32,33^ The presence of sorbent particles in solution may affect *R*_f_^e_aq_–^ by screening UV light penetration. Equation 2 illustrates the desired reaction between e_aq_^–^ and a target compound (*C_i_*). Adsorbents may impact this term by making the target compound unavailable for e_aq_^–^ through adsorption. Equation 3 describes the scavenging of e_aq_^–^ by nontarget scavengers (*S_i_*). The combined impact of all e_aq_^–^ scavengers on the PFAA reaction rate can be generalized by the pseudo-first-order scavenging capacity, *k*′_S_ (s^–1^). Carbon sorbents may themselves be important e_aq_^–^ scavengers depending on their surface chemistry; for example, higher e_aq_^–^ quenching may be expected by carbonyl functional groups.^32,34^ Sorbent properties can thus decrease steady-state e_aq_^–^ concentrations and negatively impact PFAA destructive treatment.

This study assesses the effect of adsorbent material properties on the potential to simultaneously decompose PFAAs and regenerate PFAA-laden carbon sorbents using UV/sulfite. Our objectives are to elucidate the limiting mechanisms of the PFAA reduction process for sorbents suspended in water and to determine appropriate engineering designs that can overcome these limitations. Three carbonaceous sorbents were evaluated based on their varying material properties: GAC, carbon nanotubes (CNTs), and polyethylenimine-modified lignin (lignin). GAC and CNTs are model hydrophobic adsorbents implemented to remove both long- and short-chain PFAA.^35,36^ Modified lignin is a novel adsorbent derived from agricultural waste materials with ionizable amine groups, providing it with high PFAA sorption capacity at low pH and limited sorption at high pH.^37^ Rate limiting mechanisms in the heterogeneous sorbent-water systems were elucidated by measuring the removal rates and defluorination of model long- and short-chain perfluorocarboxylic acids (PFCAs), PFOA and perfluorobutanoic acid (PFBA), in batch experiments. Reaction-limiting effects of the heterogeneous systems were further studied by quantifying e_aq_^–^ formation rates (*R*_f_^e_aq_–^) and scavenging capacity (*k*′_s_) in each suspension. Finally, to validate the quantified parameters, *R*_f_^e_aq_–^ and *k*′_s_ were used in a kinetic model to predict the degradation profile of PFOA in each heterogeneous system.

## Materials and Methods

### Materials

Sodium perfluorooctanoate (PFOA-Na, C7F15COONa, 97%) and sodium chloroacetate (MCAA, ClCH_2_COONa, 98%) were purchased from Sigma-Aldrich, Alfa-Aesar. Heptafluorobutyric acid (PFBA, C4HF7O2, >98.0%) was purchased from Tokyo Chemical Industry (TCI). Sodium sulfite (Na_2_SO_3_, >98%) was obtained from Fisher Scientific. Granular activated carbon (Filtrasorb 400, denoted as GAC) and carbon nanotubes (S-MWNT-1020, denoted as CNT) were provided by Calgon Carbon and Shenzhen Nanotech Port Co., Ltd., respectively. The polyethylenimine-modified lignin is a positively charged sorbent made from corn stover, as previously described.^37^

### Degradation Experiments

PFCA solutions in Milli-Q water were prepared at 12 μM unless otherwise expressed. Sodium sulfite (Na_2_SO_3_) was added at 20 mM. Carbon materials were added to the solution at 1 g/L concentration. The suspension was sonicated for 15 min and shaken for at least an hour. Afterward, the pH was adjusted to 10 with 1 mM solutions of sodium hydroxide (NaOH) and hydrogen chloride (HCl). The suspension was agitated for 24 h to equilibrate PFCA adsorption before starting the degradation experiments. The total volume of the solution was 500 mL.

The degradation experiment was performed in a glass photo reactor (ACE Glass, Vineland, NJ) with a 254 nm UV lamp (GPH212T5*L*/4, Heraeus Noblelight Ltd., China, 10 W) in a quartz immersion well. The outer shell of the reactor was covered with aluminum foil for safety. The reactor was placed in an ice water bath to maintain the temperature at 20 °C. The reactor was not purged with nitrogen gas to reduce any losses of volatile intermediates from the solution. Any dissolved oxygen initially in the reactor will be rapidly consumed by reaction with e_aq_^–^ and sulfite radicals,^33,38,39^ thus having a negligible quenching effect. The solution was constantly stirred by a magnetic stir bar (800 rpm) to reduce mass transfer limitations. At each time point, an aliquot of at least 8 mL containing water and particles was extracted either by pipet (for smaller CNT and Lignin particles) or poured (for larger GAC particles) into 15 mL polypropylene (PP) centrifuge tubes. Only the aqueous phase was sampled for the PFBA and MCAA experiments because there is insignificant adsorption for all particles at reaction conditions (Figure S1). Each time point represents the mean of duplicate samples unless otherwise indicated. No significant PFCA loss was measured by adsorption to the glass walls or evaporation during dark control tests without particles (Figure S2).

### Sample Preparation and Extractions

To analyze aqueous phase compounds, each sample was centrifuged (Beckman Coulter Avanti J-E, rotor: JA-10) at 6000*g* for 20 min at ambient temperature to separate the particles. The aqueous supernatant was removed via pipet and analyzed for fluoride and unadsorbed PFCA. To analyze sorbed PFCA, 10 mL of acidified methanol solution (9 mL of methanol and 1 mL of 1% acetic acid in Milli-Q) was used to extract sorbed PFCA from the recovered particles using a modified procedure from Zenobio et al.^40^ The 10 mL suspension was sonicated for 10 min then placed on a rotating mixer for at least 24 h. Then, the particles were centrifuged as mentioned, and the 10 mL supernatant was transferred to a 50 mL polypropylene (PP) tube. The extraction procedure was repeated sequentially four additional times. All five extraction solutions were combined in the 50 mL tube for analysis.

After extracting the adsorbed PFCAs, the sorbent particles recovered from each sample were dried and weighed to calculate the amount of PFCA adsorbed per particle mass in each sample. Briefly, solids were quantitatively transferred to a preweighed glass vessel and placed in a vacuum oven at 150 °C until dry. The glass vessel was then weighed again to determine the mass of solids in each sample. PFOA extraction efficiencies for GAC (88 ± 35%) and CNT (96 ± 12%) are reported in Figure S3. Errors are primarily a result of the challenge of accurately weighing small carbon masses. Given this uncertainty, four (*n* = 4) experimental replicate extractions were performed for GAC samples in each time point to ensure sound data quality and statistical weight of the results.

### Fluoride Analysis

Fluoride ion was measured using a fluoride ion selective electrode (ISE, Fisherbrand accumet Solid State Combination ISE) according to EPA method 9214. Briefly, 5 mL of the particle-free solution is mixed with 5 mL of total ionic strength adjustment buffer (TISAB) solution for a 1:1 V/V ratio in 15 mL PP beakers. While stirring (200 rpm), the ISE probe was inserted, and a measurement was taken once the voltage equilibrated after ∼3 min. The ISE was calibrated over a 0.02–30 ppm fluoride ion range before measuring a set of samples.

### PFCA and MCAA Analysis

PFCA and MCAA analysis was performed using direct injection liquid chromatography tandem mass spectrometry (LC-MS/MS) in an Agilent 1100/6430 HPLC-MS (QqQ) with electrospray ionization (Agilent Technologies). PFCAs were separated using gradient elution, detected with quantitative and qualitative ion transitions (Table S1), and quantified via external calibration. MCAA samples were separated through isocratic elution, detected through single ion mode, and quantified by external calibration. All samples were filtered through 0.2 μm cellulose acetate filters prior to analysis. Additional details on analytical methods, quality assurance, and quality control protocols are described in Text S2.

PFCA transformation products were used to understand the extent of the PFOA decomposition in suspension. A fluorine mass balance was calculated considering the fluoride atoms in detected PFCA products and fluoride ions released as described in Bowers et al.^41^ The total initial fluorine concentration is the measured total fluorine mass before particles are added. Additional details on the mass balance calculation are presented in Text S3.

### Quantifying Hydrated Electron Formation Rates (*R*_f_^e_aq_–^)

Quantification of e_aq_^–^ formation rates (*R*_f_^e_aq_–^) typically requires measuring the system’s photon flux (*I*_o_) through actinometry.^42^ However, performing actinometry in a particle suspension is impaired due to technical challenges such as the adsorption of actinometer molecules onto particles and light attenuation caused by particles. These challenges may lead to inaccurate *I*_o_ and *R*_f_^e_aq_–^ values. Therefore, *R*_f_^e_aq_–^ was determined empirically by using MCAA as a hydrated electron probe molecule because it reacts rapidly with e_aq_^–^ (*k*_MCAA_ = 1.0 × 10^9^ M^–1^ s^–1^)^21^ and does not undergo direct photolysis at 254 nm.^42^ Additionally, we confirmed that a low concentration of MCAA (10 ppm) does not adsorb significantly to the carbon particles at our reaction conditions (20 mM sulfite at pH10) through adsorption tests illustrated in Figure S4. Therefore, MCAA was only tracked in the aqueous phase, and no particle extraction was performed.

The empirical method relies on the rate law describing the disappearance of a target compound under UV/sulfite given by eq 4
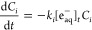
4where *k_i_* (M^–1^ s^–1^) is the bimolecular rate constant of the target compound *i* with e_aq_^–^ and [e_aq_^–^]_*t*_ (M) represents the time-dependent concentration of hydrated electrons.^32,42^ [e_aq_^–^]_*t*_ is a function of the hydrated electron formation rate over the total e_aq_^–^ consumption rate eq 5.
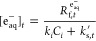
5

Note that the denominator, which represents the total e_aq_^–^ consumption rate, includes the contribution of the target compound (*k*_*i*_*C*_*i*_) and of the scavengers (*k*_s,*t*_^′^). The target compound’s observed pseudo-first-order rate constant (*k*_obs_, s^–1^) is therefore defined by eq 6.
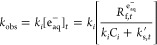
6

Notice that *R*_f,*t*_^e_aq_–^ and *k*_s,*t*_^′^ are time-dependent. For this analysis, it is assumed that the e_aq_^–^ formation rate (*R*_f_^e_aq_–^) and scavenging capacity (*k*_S_^′^) do not change appreciably over the short time intervals (between 5 and 30 min) in which the MCAA probe compound loss rate is monitored, given the excess initial MCAA concentrations (5–100 ppm) and high *k_i_* of MCAA with e_aq_^–^ (*k*_MCAA_ = 1.0 × 10^9^ M^–1^ s^–1^).^21^

Taking the inverse of *k*_obs_eq 6, yields the following linear relationship eq 7.
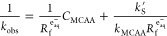
7

Equation 7 shows that *k*_obs_^–1^ (min) changes linearly with different initial probe concentrations (*C*_MCAA_), and the slope of this relationship is equivalent to the inverse e_aq_^–^ formation rate, . Thus, by plotting the inverse pseudo-first order loss rate of different initial MCAA concentrations for a given system, we can obtain the initial *R*_f_^e_aq_–^ by taking the inverse slope of the resulting regression eq 7. This approach has been used to probe reactive species in previous studies, given that the formation rate of reactive species under constant irradiation in a given system should be consistent regardless of the presence or absence of quencher or probe compounds that do not absorb the incoming light.^43^

### Spectrophotometric Analysis to Determine Sulfite Absorbance

The fraction of monochromatic light (254 nm) absorbed by sulfite (*f*_abs,SO_3_^2–^_) is given by eq 8.
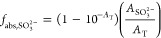
8where *A*_SO_3_^2–^_ is the light (254 nm) absorbance of 20 mM SO_3_^2–^ at pH10 without particles and *A*_T_ is the total absorbance of each particle solution. *A*_SO_3_^2–^_ and *A*_T_ were measured using a UV–vis spectrophotometer (Agilent Technologies, Cary 4000 UV–vis Spectrophotometer) in a quartz cuvette (1 cm path length). *A*_SO_3_^2–^_ was assumed to be constant with and without particles based on the traditional Beer–Lambert linear relationship, given that all measured absorbance values were <1 (Table S5). To measure *A*_T_ with particles, suspensions were shaken vigorously before transferring a 2 mL aliquot into the cuvette. The procedure was done in triplicate for CNT and Lignin particles as they had well-dispersed particles and are expected to contribute most significantly to light screening compared to GAC based on visual observations (Figure S5). Additionally, suspensions of larger GAC particles were unstable without mechanical agitation, making accurate spectrometric measurements challenging. Text S1 describes the relationship between optical parameters and the formation rate of hydrated electrons through direct photolysis of the source chemical. Hydrated electron photogeneration is also discussed extensively in the literature.^32,42,44,45^

### Sulfite and Sulfate Analysis

Changes in combined sulfite (SO_3_^2–^) and sulfate (SO_4_^2–^) concentrations were measured using a Dionex Ion Chromatograph (IC) to obtain a sulfur mass balance. Analyses were run under isocratic conditions using 20 mM KOH eluent, a suppressor at 60 mA, and a flow rate of 1.2 mL/min for a total run time of 16 min. The sample injection volume was 25 μL.

### Estimating Scavenging Capacity (*k*′_S_)

PFBA was used to probe aqueous electron scavenging because it has a relatively lower reactivity with e_aq_^–^, allowing the scavengers to have a measurable contribution to the total e_aq_^–^ consumption rate. This is a competitive kinetics experiment where a slower loss rate of PFBA indicates a greater scavenging rate of e_aq_^–^. The value of the scavenging capacity, *k*′_S_, was estimated using eq 9
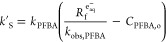
9where *k*_PFBA_ is the reported bimolecular rate constant between PFBA and e_aq_^–^ (1.28 ± 0.04 × 10^7^ M^–1^ s^–1^),^46^*k*_obs,PFBA_ is the pseudo-first-order rate constant of PFBA measured through kinetic data under each condition, and *C*_PFBA,0_ is the initial PFBA concentration (12 μM). *R*_f_^e_aq_–^ is empirically estimated using the loss rate of the MCAA probe eq 7 as previously described. Note that *k*_obs_ over the bimolecular rate constant eq 6 provide an estimate of the e_aq_^–^ concentration (M) eq 10.
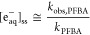
10

This assumes steady-state for e_aq_^–^ and should be valid when the target compound contributes minimally to the total hydrated electron consumption rate, such as for the slow-reacting PFBA. Inserting eq 10 into eq 9 provides the following relationship to quantify *k*′_S_.
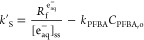
11

## Results and Discussion

### Adsorbent Properties

Three adsorbents (GAC, CNT, and lignin) were selected for the study because of their different properties, as shown in Table 1. GAC and CNT capture compounds from water primarily through hydrophobic interactions. On the other hand, modified lignin relies on the electrostatic attraction of anionic compounds to its positively ionized quaternary amines, thus resulting in a material with a high pH point of zero charge (PZC) (Table 1).

**Table 1:** Adsorbent Material Properties

adsorbents	BET specific surface area (m^2^/g)	pH_PZC_
GAC	861	7.2–8.6^47,48^
CNT	147	6.5^49^
modified lignin	5.4	9.25^37^

**Figure 1:**
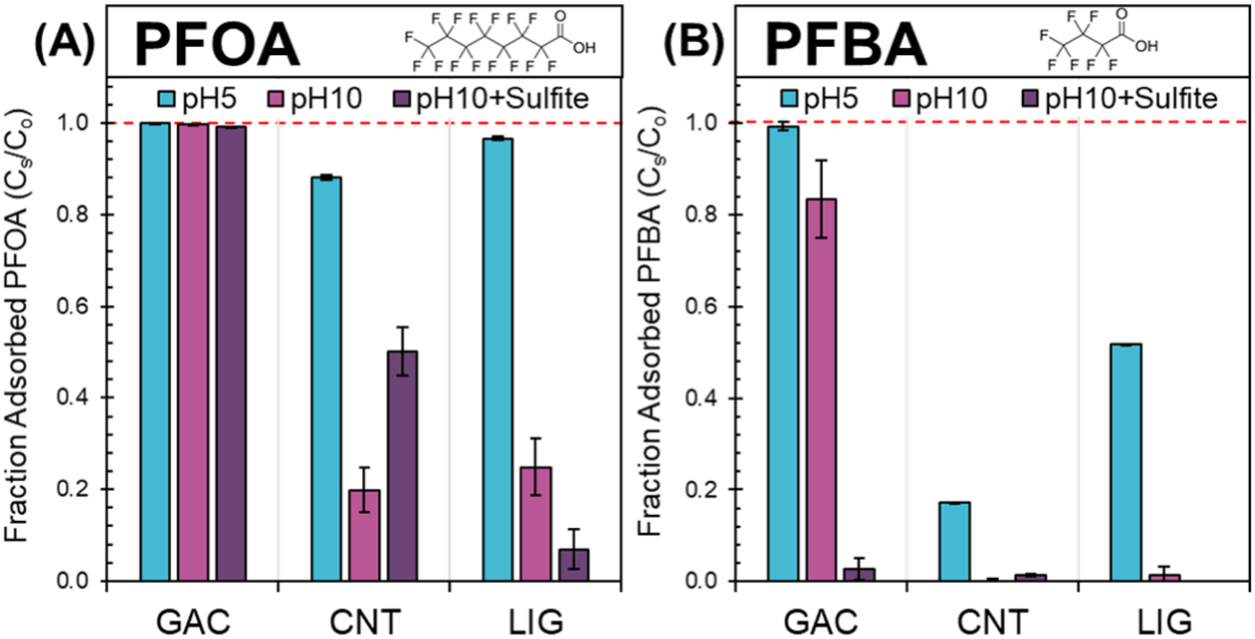
Adsorbed fraction (*C*_S_/*C*_o_ = 1 – *C*_w_/*C*_o_) of 12 μM (*C*_o_) (A) PFOA and (B) PFBA on 1 g/L of GAC, CNT, and modified lignin (LIG) in solutions of Milli-Q water at pH5, pH10, and pH10 with 20 mM sulfite (SO_3_^–2^). Aqueous phase samples were measured after 24 h of adsorption. Dashed reference lines indicate 100% sorbed mass fraction. Error bars represent the standard error of experimental duplicates of adsorption.

The sorption mechanisms and performance of these materials were assessed through batch adsorption of 12 μM of PFOA and PFBA at pH5, pH10, and in the presence of 20 mM sulfite (SO_3_^–2^) at pH10. Both pH5 and pH10 were selected to evaluate adsorption below and above the PZC of the three materials (Table 1). Figure 1 shows the PFOA and PFBA adsorbed fraction to each material for each condition. PFOA adsorbs strongly (>99.8%) to GAC at pH5 and pH10, likely because pH-independent hydrophobic sites are abundant. With sulfite at pH10, adsorption of PFOA was relatively unchanged (99.122 ± 0.004%), confirming the predominance of hydrophobic interactions. For CNTs, high PFOA adsorption is measured at pH5 (88.1 ± 0.6%), but only 20% was adsorbed at pH10. The repulsion of anionic PFOA from CNTs’ net negative surface charge at pH10 may explain the decrease in adsorption and suggest that electrostatic interactions account for a part of the sorption on CNTs.^50^ PFOA adsorption on CNTs increased to 50 ± 5% when adding 20 mM SO_3_^–2^ at pH10, potentially due to both salting out and screening of electrostatic repulsions.^51,52^ For lignin, PFOA adsorption was 96.7 ± 0.3% at pH5, but decreased to 25 ± 6% at pH10 without SO_3_^–2^ and to 7 ± 4% with SO_3_^–2^ at pH10. This demonstrates lignin’s ability to bind PFOA at low pH through Coulombic attraction and its potential for easy regeneration at basic pH. Additionally, it suggests that competitive adsorption of the inorganic divalent anion (SO_3_^–2^) decreases PFOA adsorption. Short-chained PFBA is adsorbed well on GAC (99.3 ± 0.7%) at pH5 but decreased at pH10 (83 ± 8%). Adsorption is further hindered at pH10 with SO_3_^–2^ (3 ± 2%), suggesting that competition for adsorption sites with divalent inorganic anions is an important effect on PFBA sorption onto activated carbon.^47,53^ Due to CNTs’ lower surface area (Table 1), PFBA sorbs poorly at pH5 (17.3 ± 0.1%) and not at all at pH10, both without and with sulfite. For lignin, PFBA showed better sorption than CNTs at pH 5 (51.67 ± 0.03%) but also had no sorption at pH10 without and with SO_3_^–2^. These adsorption measurements confirmed the expected interactions between model long- (PFOA) and short- (PFBA) chained PFCAs with the three adsorbent materials. The following sections explore how the different partitioning of PFCAs to these adsorbents impact their degradation kinetics and defluorination by hydrated electrons.

### PFOA Degradation with e_aq_^–^ in Sorbent Particle Suspensions

**Figure 2:**
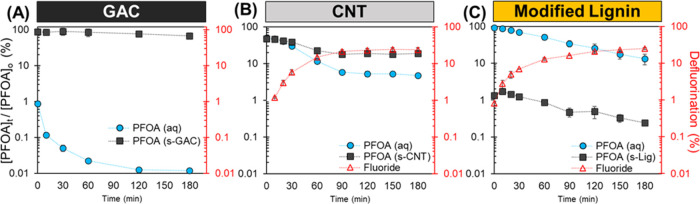
PFOA percent concentration (left *y*-axis) and percent defluorination (right *y*-axis) during the reaction with 20 mM sulfite irradiated under 254 nm UV light at pH10 in the presence of 1 g/L carbon sorbents (A) GAC, (B) CNT, or (C) Modified Lignin. Note that the vertical axis is on a logarithmic scale. Initial PFOA concentration before the addition of particles ([PFOA]_o_) was 12 μM, represented as 100%. PFOA was monitored in the aqueous phase (aq) and extracted from the sorbent (s). Error bars represent the standard error of replicate samples (*n* = 2 for all and *n* = 4 for sorbent phase PFOA in GAC). Fluoride was not detected (LOD = 0.02 ppm F^–^) when PFOA was reacted with GAC present.

Before initiating the photoreaction (254 nm), PFOA was preabsorbed on the three particle suspensions at pH10 with 20 mM sulfite for at least 24 h. Each suspension enabled different amounts of adsorbed and dissolved PFCA at these conditions (Figure 1). The data presented in Figure 2 and the subsequent analysis evaluate the extent of PFOA partitioning, degradation, and defluorination for each suspension over time. Fluorine mass balances including detected shorter chained PFCA products in the solid and aqueous phases are reported in Figures S6 and S7.

#### Granular Activated Carbon (GAC)

The change of PFOA mass in both the aqueous and adsorbed phases in the presence of GAC particles is shown in Figures 2A and S6A. As discussed, most PFOA is strongly adsorbed onto GAC through hydrophobic interactions,^35^ while only ∼1% of the total initial PFOA mass is present in the aqueous phase before the reaction begins. Hydrated electrons quickly reduce the PFOA mass in the aqueous phase once the reaction starts, while the abundant adsorbed PFOA mass does not change significantly (Figures 2A and S6A). The slight decrease in sorbed PFOA mass may be from desorption into the aqueous phase, but this amount is limited over the time scale of the measurement. The absence of measurable fluoride ions and a low detected amount of perfluoroheptanoic acid (PFHpA, C7) (Figure S7A) are consistent with the limited PFOA loss measured. Note that PFHpA is present as an impurity at *t* = 0 (0.35 ± 0.03% of the F-mass balance), so measured excess PFHpA may be formed from the transformation of the aqueous phase PFOA (Figure S7A). These results indicate that PFOA degradation in a GAC suspension occurs rapidly in the aqueous phase. However, adsorbed PFOA is not desorbing fast enough and remains unreactive over the experiment’s time scale. Therefore, adsorbed PFOA could have a significantly lower probability of encountering e_aq_^–^. A potential explanation could be that hydrated electrons formed in the bulk homogeneous solution^54^ cannot reach PFOA adsorbed in the internal GAC pores.^55−57^ Additionally, 254 nm light penetration into the particle pores may be limited, leading to lower sulfite photolysis in the pore water and, therefore, decreased e_aq_^–^ formation within the sorbent. Furthermore, any hydrated electrons generated within the pore water may be quenched by the GAC surface faster than it is reacting with the adsorbed PFOA. These mechanisms are explored in detail later in the manuscript.

#### Carbon Nanotubes (CNTs)

CNTs have a lower adsorption capacity than GAC at reaction conditions, so approximately 50% of the initial PFOA mass resides in the aqueous phase (Figure S6B). Hence, the reaction is hypothesized to proceed to a greater extent as a higher PFOA mass fraction is available for e_aq_^–^ attack in the bulk aqueous phase. PFOA decreases in both the aqueous and solid phases as the reaction proceeds until there is no further measurable reduction after 90 min (Figure 2B). The remaining 25% of the total PFOA mass was distributed between the CNTs and aqueous phase at a 4:1 ratio, substantially less than the 1:1 ratio at the beginning of the experiment (Figure S6B). This suggests that the PFOA reaction rate in the aqueous phase is faster than the desorption rate or the reaction on the solid phase. Assessments quantifying desorption rates of PFOA from CNTs and GAC showed that aqueous phase disappearance is indeed controlled by the desorption rate (Figure S8), which supports this hypothesis.

After 3 h of reaction, 24 ± 3% defluorination was achieved (Figure 2B), and shorter-chain PFCAs were detected (Figure S7B). PFHpA (C7) increases over the reaction time, and after 90 min, shorter PFCAs (C6, C5, and C4) are detected. Most short-chained products are found in the aqueous phase because of their higher water solubility.^58^ The limited sorption of the short-chained PFCAs may also be exacerbated by the net negative surface charge of CNTs at pH10, which repels anionic headgroups and competition with PFOA and inorganic anions (SO_3_^–2^) for adsorption sites.^47^

The observed decrease in sorbed PFOA mass may be due to degradation on the surface or desorption into the aqueous media. To determine whether PFOA adsorbed onto CNTs is amenable to hydrated electron attack, a test was performed at a lower initial PFOA concentration (1.2 μM) so that ∼90% is initially adsorbed onto CNTs. Figure S9 illustrates that the adsorbed PFOA mass remains practically unchanged as the reaction proceeds. Moreover, no fluoride or short-chained PFCAs were detected, likely because they were generated below their detection limits (Table S1 for PFCA and 0.02 ppm for F^–^). Similar to GAC, these results support the hypothesis that adsorbed PFOA is unreactive to e_aq_^–^ on the CNT surface at these reaction conditions and time scales. Furthermore, the slower aqueous phase reaction observed for PFOA in the presence of CNTs suggests that these particles may also negatively impact decomposition rates by decreasing the available e_aq_^–^ steady-state concentration. This is discussed in the following sections when determining the e_aq_^–^ formation and consumption rates in the presence of particles.

#### Modified Lignin

As described previously, modified lignin is engineered to adsorb PFCAs at low pH, where its positively charged amines capture negatively charged PFCAs but repel them at higher pH and ionic strength (Figure 1). Therefore, at our reaction conditions (pH10 and 20 mM sulfite), most of the PFOA (>90%) is in the aqueous phase (Figures 2C and S6C). Because only ∼1.3% PFOA is adsorbed on the lignin, the reaction with e_aq_^–^ is expected to be faster and achieve higher defluorination than with GAC and CNTs. The PFOA concentration steadily decreases with time, simultaneously releasing fluoride (Figure 2C). Pseudo-first-order kinetics in both phases were similar, suggesting that desorption of PFOA from lignin was not rate limiting. Unlike CNT, aqueous C7 was the only detectable PFCA product (Figure S7C). Moreover, the pseudo-first-order rate constant for PFOA in lignin suspension (0.011 min^–1^) is significantly lower than the particle-free reaction (0.080 min^–1^) (Figure S10 and Table S2). About 25.1% defluorination was achieved after 3h for lignin, similar to CNT (24% defluorination), despite most PFOA being initially in the aqueous phase (Figure 2B,C). This result indicates that similar to CNT, lignin particles slow the aqueous phase reaction rate beyond the effects of adsorption.

### Impact of Particles on Reactivity beyond Adsorption

**Figure 3:**
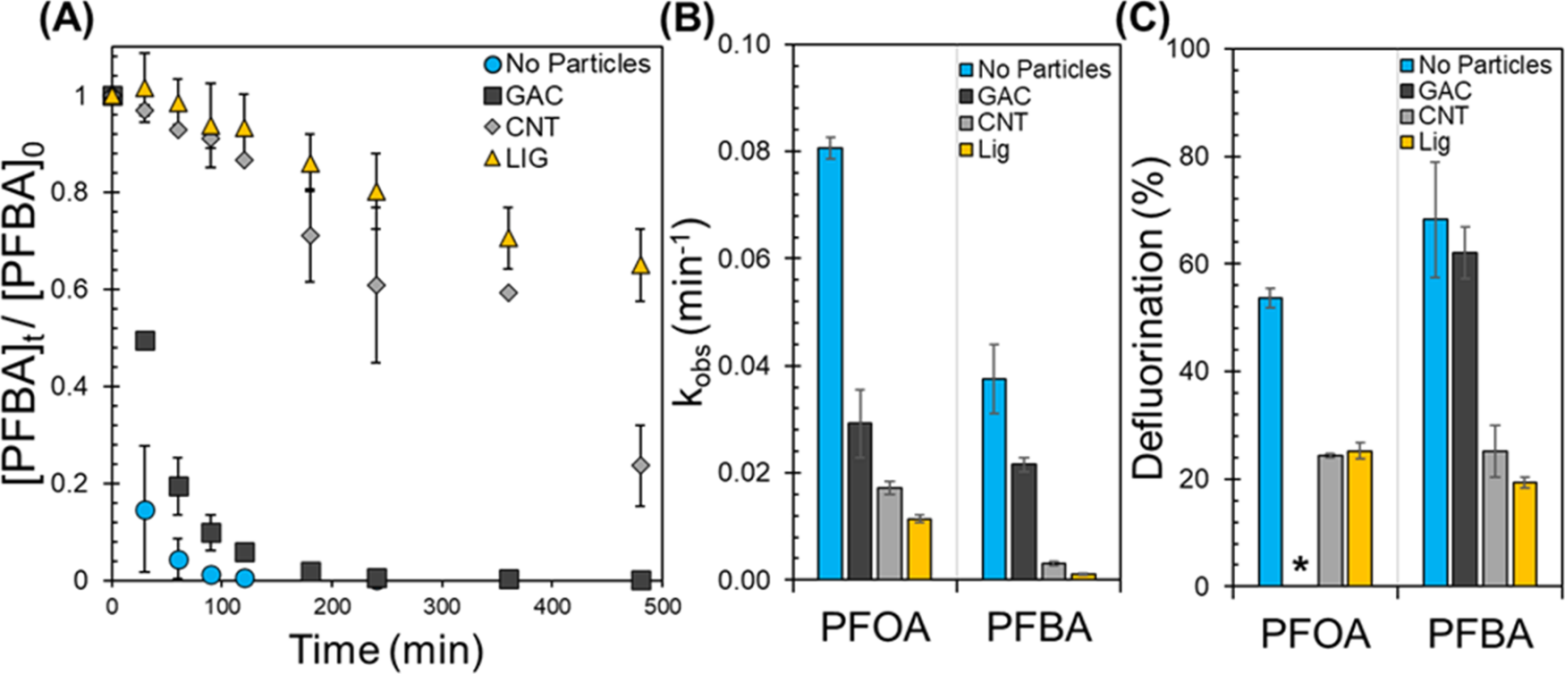
(A) PFBA decomposition with 20 mM SO_3_^–2^ and 254 nm UV light at pH10, without and with 1 g/L particles (GAC, CNT, LIG). [PFBA]_o_ = 12 μM. Error bars represent the standard error of replicate measurements (*n* = 3 for CNT, *n* = 2 for the rest). (B) Observed pseudo-first-order rate constants (*k*_obs_) for PFOA and PFBA (12 μM initial concentration) without and with particles. *k*_obs_ for PFOA in the presence of GAC was measured from the aqueous PFOA disappearance. For the rest, *k*_obs_ were measured from the total disappearance profile of each compound, thus the sum of the aqueous and solid phases. (C) Percent defluorination for PFOA at *t* = 180 min and PFBA at *t* = 480 min without and with particles. *Fluoride was not detected (LOD = 0.02 ppm F^–^) when PFOA was reacted with GAC.

To better understand the impact of the carbon particles on aqueous phase PFCA degradation with hydrated electrons, PFBA degradation kinetics and defluorination were measured for each suspension and compared to PFOA. PFBA does not significantly adsorb for the three particles at the reaction conditions (20 mM SO_3_^–2^, pH10, and 1g/L particles) (Figure 1B). Hence, it is possible to evaluate the impact of reaction-limiting mechanisms (e.g., light attenuation, scavenging) using PFBA. Figure 3A shows the degradation profile of PFBA in the absence and presence of the particles. PFBA degradation kinetics are significantly impacted by the presence of the particles despite not being adsorbed (Figure S11). Even though PFBA appears less reactive than PFOA with e_aq_^–^,^32^ the change in *k*_obs_ for both PFOA and PFBA degradation with each sorbent follows the same trend: No particles > GAC > CNT > Lignin. This trend suggests that the particles are decreasing PFCA decomposition rates by affecting the steady-state concentration of e_aq_^–^ to different degrees.

The percent defluorination at the end of the reaction (Figure 3C) provides additional insight into activity differences. No fluoride ion was detected for PFOA on GAC because most PFOA mass was adsorbed and thus unavailable for reaction with e_aq_^–^. Conversely, most PFBA mass was not adsorbed to the GAC (Figure 1B), yielding a similar percent defluorination to the control without particles (Figure 3C). This result reinforces the hypothesis that the target contaminant must be in the aqueous phase to react with the e_aq_^–^, as this is where the reactive species are generated and reaction amenable sites (e.g., α carbon to the carboxylic head)^59−61^ are more exposed to e_aq_^–^. Figure 3C also shows that the defluorination observed in CNT and lignin suspensions were similar for PFOA (24 ± 3% for CNT and 25 ± 2% for lignin) and for PFBA (25 ± 5% for CNT and 19 ± 1% for lignin) despite the significant difference in the solid–water mass distribution of both model PFCAs (Figure 1). Hence, sorption alone is insufficient to explain the particles’ impact on PFCA degradation kinetics and defluorination by hydrated electrons. The following section quantifies the hydrated electron formation rates (*R*_f_^e_aq_–^), scavenging capacities (*k*′_S_), and steady-state concentrations to explain these differences.

### Quantifying Hydrated Electron Formation Rates (*R*_f_^e_aq_–^)

As described in Text S1, *R*_f_^e_aq_–^ depends on the system’s photon flux (*I*_o_), the concentration of the hydrated electron source chemical (SO_3_^2–^), the total amount of light absorbed by the source chemical, and the quantum yield for producing hydrated electrons. Here, MCAA was used as a probe molecule to empirically quantify hydrated electron formation rates using eq 7 by measuring the loss rate of nonadsorbing (Figure S4) and highly e_aq_^–^ reactive (*k*_MCAA_ = 1.0 × 10^9^ M^–1^ s^–1^)^21^ MCAA in each particle system.

**Figure 4:**
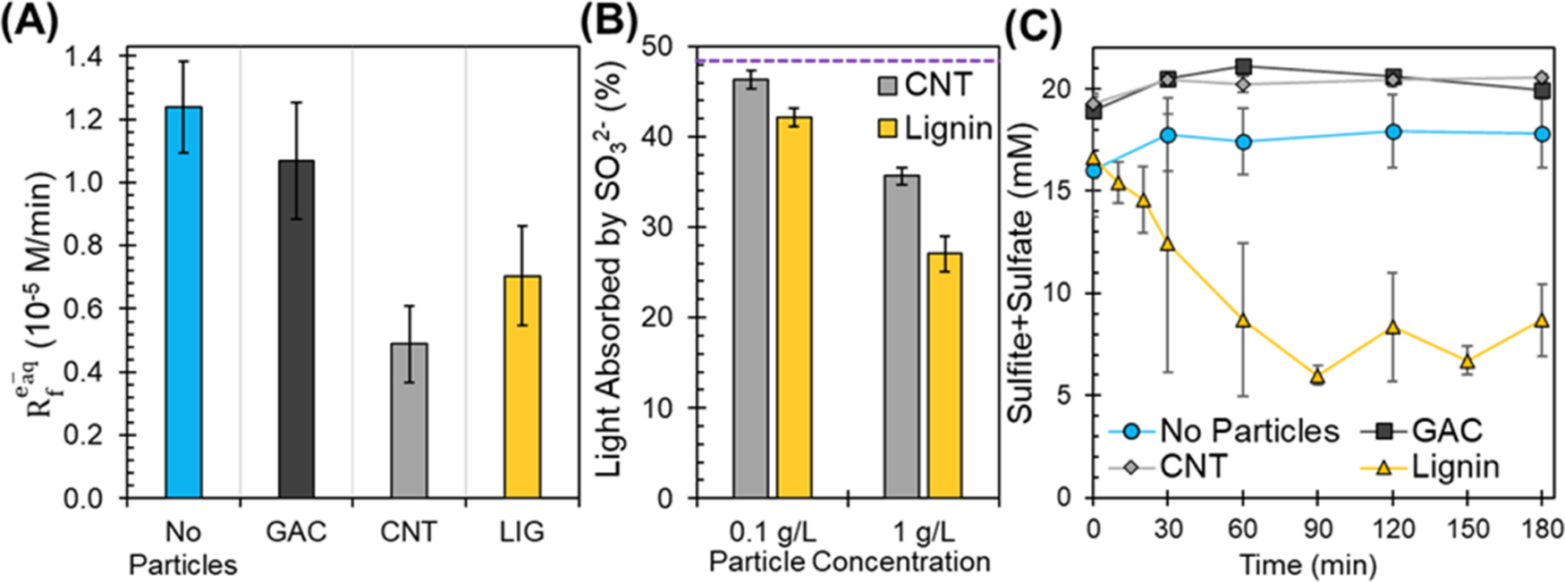
(A) Mean hydrated electron formation rates (*R*_f_^e_aq_–^) of each system calculated from the slope of the inverse MCAA pseudo-first-order rate constant (*k*_obs_^–1^, min) as a function of initial MCAA concentration from Figure S12. Error bars represent the standard error from the slope regression. (B) Percent of total light (254 nm) absorbed by SO_3_^2–^ (20 mM) at pH10 for different CNT and lignin concentrations measured through spectrophotometry. The dashed purple line indicates the percent light absorbed by 20 mM SO_3_^2–^ at pH10 without particles. (C) Combined sulfite and sulfate (SO_3_^2–^ + SO_4_^2–^) concentration (mM) with time of UV exposure in PFOA degradation experiments. Initial sulfite concentration is 20 mM and PFOA concentration of 12 μM at pH10 with 1 g/L particles. Error bars represent standard errors of experimental duplicates.

The linear relationship between the inverse pseudo-first-order rate constant (*k*_obs_^–1^) and the initial concentration of MCAA eq 7 for each system yields a slope of 1/*R*_f_^e_aq_–^ (Figure S12). Statistical parameters of the regressions are reported in Table S3. Empirically estimated hydrated electron formation rates are shown in Figure 4A. The presence of each particle affected *R*_f_^e_aq_–^ differently: No particles ≥ GAC (14 ± 18% decrease) > lignin (47 ± 16% decrease) ≥ CNT (61 ± 11% decrease). Interestingly, the e_aq_^–^ formation rate was higher for lignin than CNT, even though lignin yielded lower *k*_obs_ for PFCA degradation compared to the other sorbents (Figure 3B). This suggests that lignin may be scavenging e_aq_^–^ at a higher rate than CNTs, resulting in a lower steady-state e_aq_^–^ concentration and lower reaction rates for both PFOA and PFBA.

Particles may affect hydrated electron formation rates by decreasing the available light for sulfite absorption (eq 1) through light screening. The absorption of 254 nm light by solutions without and with CNT or lignin particles was measured to estimate the fraction of available light absorbed by SO_3_^2–^ (*f*_abc,SO_3_^2^_^_–_^) using eq 8 and to calculate its contribution to decreasing e_aq_^–^ formation rates. The assessment was performed on CNT and lignin particle suspensions, given that these particles showed the most significant changes to *R*_f_^e_aq_–^ (Figure 4A). GAC did not significantly affect *R*_f_^e_aq_–^compared to the no-particle case (Figure 4A) and was therefore not assessed. Visual observations also show that the GAC suspension allows more photons to pass through the solution (Figure S5). The percentage of light absorbed by sulfite in CNT and lignin suspensions is shown in Figure 4B. Without particles, the sulfite solution absorbs 48.5% of the incoming light (dashed line in Figure 4B). As particles are added, the total absorbance increases (Table S5), indicating that the particles absorb or scatter light. However, the estimated fraction of light absorbed by SO_3_^2–^ decreases in the presence of these particles (Figure 4B). Both particle size and surface chemistry can affect their ability to absorb light. For example, CNTs’ graphene structure and surface oxygen-containing groups may undergo direct photolysis by 254 nm photons.^62,63^ Lignin is a well-known chromophore responsible for the light absorbance of aquatic DOM,^64^ and lignin’s aromatic methoxy groups can undergo direct photolysis at 254 nm.^65^ At 1 g/L of particles used in the degradation experiments, the amount of light absorbed by sulfite decreased to 35.6% with CNT and 27.0% with lignin (Figure 4B). Only considering the parameters described in Text S1 and assuming *I*_o_ is constant, the resulting percent difference in e_aq_^–^ formation rates is estimated by comparing *f*_abc,SO_3_^2–^_ with 1 g/L particles over the no-particle case (Figure 4B). The calculation estimates that e_aq_^–^ formation would decrease by 27% with CNTs and 44% with lignin as less light is absorbed by the e_aq_^–^ source chemical (SO_3_^2–^). Although the decrease in SO_3_^2–^ light absorption is consistent with the *R*_f_^e_aq_–^ measurements using the MCAA probe, the differences in e_aq_^–^ formation rates for these particles (decreases of 61% with CNT and 46% with lignin) are not fully accounted for by the decrease in light absorption mechanism alone, especially for CNTs. Particle’s impact on photon flux may also contribute to the observed decrease in *R*_f_^e_aq_–^.

Consumption of sulfite also affects e_aq_^–^ formation rates.^42^ Sulfite radicals (SO_3_^•–^) are generated during sulfite (SO_3_^2–^) UV photolysis eq 1, which are then expected to oxidize into sulfate (SO_4_^2–^).^32,66^ Sulfite radicals may also be consumed by other components in suspensions. As an auxiliary assessment, the combined mass of SO_3_^2–^ and SO_4_^2–^ was measured for each system to monitor the expected total sulfur mass with reaction time. Results shown in Figure 4C demonstrate that the sulfur balance is unchanged for No Particles, GAC, and CNT. However, the sulfur mass balance decreases in the presence of lignin particles, suggesting that lignin is inducing the transformation of SO_3_^2–^ into species other than SO_4_^2–^. Sulfite can transform into other terminal ions (e.g., S_2_O_8_^2–^, S_2_O_6_^2–^, SO_5_^2–^) depending on the water components through radical chemistry.^39,66,67^ Moreover, sulfite may react with lignin functional groups, leading to sulfonation of the material.^68,69^ Consumption of SO_3_^2–^ through these potential mechanisms would eventually affect e_aq_^–^ formation rates. This finding has broader implications for implementing UV/SO_3_^2–^ treatment as the presence of DOM in natural waters, which may contain lignin-like components, could similarly transform SO_3_^2–^ under UV irradiation and impact the efficiency of the advanced reduction process. More research is needed to identify SO_3_^2–^ terminal products in such cases and determine their effects on the treatment.

### Estimating Hydrated Electron Scavenging Capacity (*k*′_S_)

**Figure 5:**
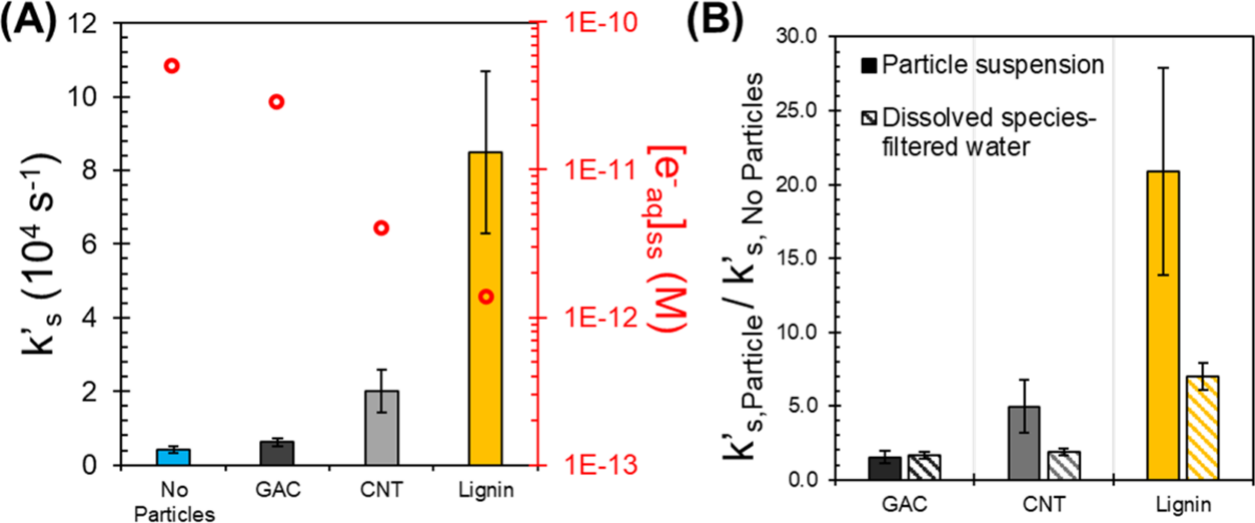
(A) Bars represent mean hydrated electron scavenging capacities (*k*′_S_) without and with 1 g/L of particles during UV/sulfite treatment calculated with eq 11 (left *y*-axis). Error bars represent the propagated standard error. Red circular markers represent the steady-state hydrated electron concentration ([e_aq_^–^]_ss_) for each system estimated from PFBA kinetic data and bimolecular rate constant using eq 10 (right *y*-axis). (B) Ratio of hydrated electron scavenging capacities of particle exposed samples (*k*′_s,Particle_) over no particle controls (*k*′_s,No Particles_). The solid colored bars (Particle suspensions) represent the *k*′_s,Particle_/*k*′_s,No Particles_ ratio for each particle suspension shown in part (A). The striped colored bars (Dissolved species-filtered water) represent the estimated *k*′_s,Particle_/*k*′_s,No Particles_ ratio of dissolved species from the filtered water of the UV/sulfite exposed suspensions discussed in Text S5. Error bars represent the propagated standard error.

Scavenging of e_aq_^–^ by the carbon sorbents may also affect PFCA degradation. It is hypothesized that sorbents with abundant electron-withdrawing functional groups will exhibit higher scavenging capacities (*k*′_S_).^70^ Modified lignin, by design, has diverse functional groups (e.g., amines, methoxy, carbonyl) and may act as a potent e_aq_^–^ scavenger. Using the most recent literature value for PFBA’s bimolecular rate constant with e_aq_^–^ (*k*_PFBA_ = 1.28 ± 0.04 × 10^7^ M^–1^ s^–1^)^46^ and our measured *k*_obs,PFBA_ for each condition (Figure 3B), [e_aq_^–^]_ss_ was estimated with eq 10 and scavenging capacities were then calculated with eq 11, which accounts for the changes in e_aq_^–^ formation rates. A sample calculation is provided in Text S4. Weight-normalized particle bimolecular rate constant with hydrated electrons (*k*_particles_, L g^–1^ s^–1^) are reported in Table S8. Results shown in Figure 5A illustrate the following trends for hydrated electron scavenging capacities: No Particles ≤ GAC < CNT < Lignin. Figure 5A also clearly shows the inverse relationship between *k*′_S_ and [e_aq_^–^]_ss_. These results support the hypothesis that any hydrated electron generated in the particle pores can be scavenged by the surface, thus leading to a decreased probability of encountering and reacting with sorbed PFCA. As hypothesized, lignin had the highest scavenging capacity, likely because lignin has ∼5% carbonyl carbons,^71^ which are proposed to be potent e_aq_^–^ quenchers.^34^ Because of its structural diversity, lignin may also contain other e_aq_^–^ quenching functional groups that significantly contribute to the material’s scavenging capacity.^61,70^

GAC and CNT significantly differ in their scavenging capacities (Figure 5A and Table S8) despite both being primarily composed of graphene. The variances are likely related to differences in their surface oxygen groups (e.g., carbonyl) that may react with e_aq_^–^.^32,34^ Furthermore, CNTs’ finer dimensions (nanometers) compared to GAC (∼500 μm) may allow more CNT surface to be in contact with the bulk solution and thus more likely to react with e_aq_^–^. More research is needed, however, to identify the functional groups that contribute most to e_aq_^–^ quenching for these materials.

Particles reacting with e_aq_^–^ may generate dissolved species (e.g., carbonate species and low molecular weight organics) that could also contribute to the suspension’s overall scavenging capacity.^34,72^ To test this hypothesis, the particle suspensions and a control solution without particles were exposed to the UV/sulfite treatment for 3 h without PFCAs. These were then vacuum filtered (Whatman grade 40 filter paper, 8 μm pore size) to remove the particles. The clear filtered water was spiked with PFOA (12 μM) to track its degradation kinetics under UV/sulfite for 1 h (Figure S13A). Results showed a similar trend in kinetics with *k*_obs_ values as follows: No Particles > GAC ≥ CNT > Lignin (Figure S13B). This result shows that generated dissolved species could contribute significantly to the scavenging capacity of each suspension. The dissolved species contribution to the overall suspension scavenging capacity was roughly estimated by assessing the ratio between the scavenging capacity of the filtered particle suspension exposed to UV/sulfite and the no particle control (details in Text S5). With this relationship, we can compare the dissolved species’ relative contributions to each particle suspension’s overall scavenging capacity (Figure 5B). It is estimated that dissolved species may account for all of GAC’s measured scavenging capacity, while CNTs and lignin may account for up to 38 and 34%, respectively (Figure 5B). Further research is needed to identify the released dissolved compounds responsible for e_aq_^–^ quenching.

Hydrated electrons reacting with adsorbents may modify the carbon surface, reducing them and affecting their reusability.^73^ To evaluate changes in adsorbent properties, PFOA and PFBA (*C*_o_ = 12 μM) adsorption was measured after 24 h at pH 5 on UV/sulfite exposed (irradiated for 3 h) particles (Figure S14). PFOA adsorption onto UV/sulfite exposed GAC and lignin remained the same; however, adsorption decreased significantly for CNTs (41% decrease) (Figure S14A). PFBA adsorption on UV/sulfite exposed CNTs also decreased significantly (62% decrease), while small changes in adsorption were observed for exposed GAC (2% decrease) and lignin (5% increase) (Figure S14B). We expected that the reduced CNT surface would enhance PFOA adsorption by increasing hydrophobic sites; however, the decrease in adsorption for both model PFCAs instead suggests that structural changes resulted in a decrease of accessible surface area due to increased CNT aggregation after UV/sulfite exposure.^62,74^ Additionally, we hypothesized that lignin would decrease its adsorption performance after UV/sulfite exposure because its aminated anion exchange sites may be reacting with hydrated electrons. Nevertheless, lignin regained its capacity for adsorbing both model PFCAs after UV/sulfite exposure and a pH swing (10 to 5) with negligible differences in adsorption, suggesting that the ionizable amine groups in modified lignin are not consumed in the process. This indicates that modified lignin could be an ideal sorbent candidate for regeneration and reuse after UV/sulfite. Further characterization of the UV/sulfite exposed carbon materials is needed to explain the observed trends and to assess the extent of adsorbent reusability.

### Kinetic Modeling of PFOA Degradation

A kinetic model was generated using the parameters (*R*_f_^e_aq_–^ and *k*′_S_) quantified during the study to corroborate if these capture the relevant phenomena controlling PFCA degradation kinetics that could be used to engineer sorbent regeneration systems following a similar approach to previous studies.^42,75^Equation 6 was used to model PFOA degradation under UV/sulfite in the presence of the different sorbents. A sample calculation is provided in Text S6. The model assumes that *R*_f_^e_aq_–^ and *k*′_S_ are constant during the reaction time. This assumption is valid over the initial reaction times but becomes less accurate as the reaction proceeds because the e_aq_^–^ source chemical (SO_3_^2–^) and quenchers are consumed.^42^ Reported bimolecular rate constants for PFOA with e_aq_^–^ vary widely (Table S7).^32^ Using the most recent value reported by Maza et al.^76^ resulted in an overestimation of PFOA kinetics in all cases (Figure S15). Instead, the best approximation to the experimental data came from the average of the two lowest reported values (*k*_PFOA,average_= 3.40 × 10^7^ M^–1^ s^–1^).^32,77,78^ This bimolecular rate constant (*k*_PFOA,average_) was selected for the predictive model.

**Figure 6:**
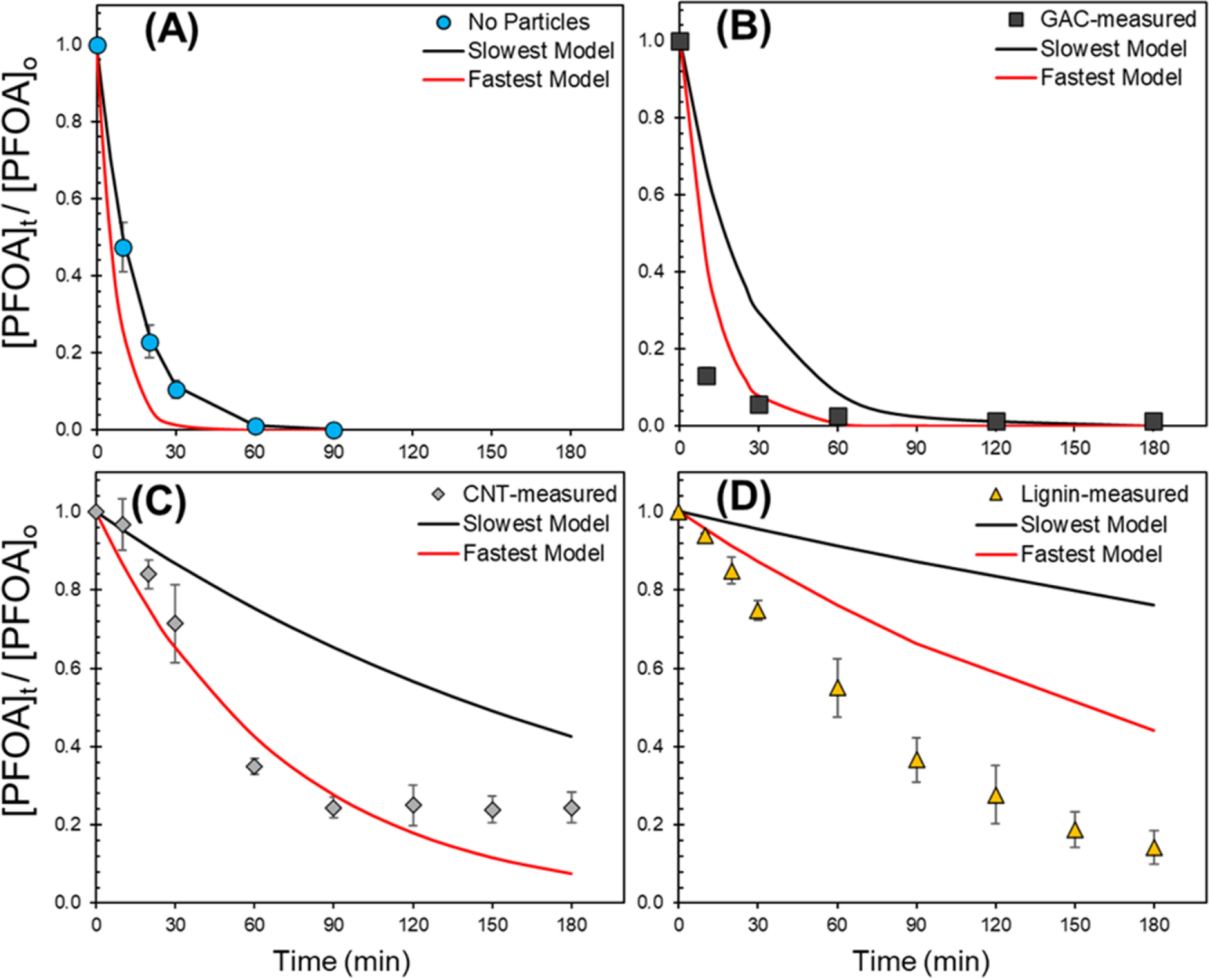
Modeling PFOA degradation in UV/SO_3_^–2^ with estimated values of hydrated electron formation rate (*R*_f_^e_aq_–^) and scavenging capacity (*k*′_S_) in (A) No Particles, (B) GAC, (C) CNT, and (D) Lignin systems. The markers illustrate measured total (aqueous + sorbed) PFOA degradation. Black and red lines represent the lower (lowest generation *R*_f_^e_aq_–^, highest scavenging *k*′_S_) and upper (highest generation *R*_f_^e_aq_–^, lowest scavenging k’_S_) bounds on the kinetic models respectively based on the standard error measured for these parameters (*R*_f_^e_aq_–^ and *k*′_S_) for each system (No Particles, GAC, CNT, and Lignin). Experiments were run at pH 10 with 20 mM initial SO_3_^–2^ and [PFOA]_0_ = 12 μM (*for GAC, [PFOA]_0,aq_ = 0.1 μM), irradiated with 254 nm light at ambient temperature (20 °C). The bimolecular rate constant *k*_PFOA_= 3.40 × 10^7^ M^–1^ s^–1^ is an average from reported literature values from Huang et al. (5.10 × 10^7^ M^–1^ s^–1^)^77^ and Szajdzinska-Pietek et al. (1.70 × 10^7^ M^–1^ s^–1^).^78^ Markers are the means of experimental duplicates, and error bars represent their standard error. *In the GAC system (B), measured PFOA concentrations are only from the aqueous phase ([PFOA]_0,aq_ = 0.1 μM), thus excluding adsorbed PFOA.

Predicted (lines) and measured (markers) PFOA degradation profiles in the absence and presence of sorbents are shown in Figure 6. The fastest (red) and slowest (black) modeled degradation profiles are based on the standard error of the parameters *R*_f_^e_aq_–^ and *k*′_S_ listed in Table S10. Tabulated values of the model results are reported in Tables S11 and S12. For the No Particle case, the data agrees with the lower bound of the estimated reactivity profile (black). For GAC, the aqueous phase data initially follows the upper bound of the modeled reactivity profile (red) but slows at longer time scales as it transitions to a desorption rate-limited process. It is important to note that the GAC model simulates PFOA aqueous phase disappearance rather than total mass loss. For CNTs, total PFOA mass loss initially follows the fastest modeled profile (red). However, as the aqueous phase PFOA depletes, the model fails to capture the transition to a desorption-controlled process at lower aqueous phase concentrations after *t* = 90 min. For lignin, the measured degradation rate is higher than the upper bound of the modeled values. The reasons for this are unclear, but it is likely related to the assumption that the parameters *R*_f_^e_aq_–^ and *k*′_S_ are constant. Lignin’s scavenging capacity is expected to decrease with time as the reactive functional groups in the particle are spent, thus allowing more e_aq_^–^ to be available for PFOA degradation and accelerating the kinetics.^42^ Lignin’s chromophores may also be consumed under UV irradiation, thus allowing more available photons for e_aq_^–^ generation. Moreover, previous studies have suggested that PFOA reactivity with e_aq_^–^ may vary depending on PFOA aggregation,^79,80^ dispersion,^81^ and interactions with e_aq_^–^ source chemicals.^24^ The presence of lignin may induce some of these molecular effects on PFOA, thus influencing its activity with e_aq_^–^. The compounding of these effects may have resulted in the observed increase in PFOA activity with lignin.

These models show that, within the experimental errors, this study captured the relevant reaction-limiting phenomena controlling PFCA degradation under heterogeneous UV/sulfite in most systems during short time scales (<90 min) by quantifying e_aq_^–^ formation and consumption rates. However, temporal changes to these parameters need to be studied further, especially for particle suspensions that exhibit high light attenuation and quenching (e.g., lignin). Implications of these findings for engineering sorbent regeneration systems are discussed in the implications section below.

## Conclusions and Environmental Implications

Hydrated electron (e_aq_^–^) advanced reduction processes (ARPs) offer a promising alternative to destroy persistent pollutants like PFAS in aqueous media. However, for ARPs to be feasible, the processes should operate in concentrated matrices, such as to regenerate spent adsorbents. This study evaluates the aspects that influence the kinetics of e_aq_^–^ ARPs in heterogeneous sorbent-PFCA systems and informs design strategies that can overcome these process challenges. The results indicate that the UV/sulfite decomposition of PFCA on carbonaceous sorbent materials is hindered by (a) PFCA sorption, (b) screening of light by the sorbent particles, (c) e_aq_^–^ scavenging by sorbent particles, and (d) in some cases the sorbent’s capacity to consume the chemical source of e_aq_^–^.

Adsorbent material properties and surface chemistry dictate which limiting factors will have a greater impact on PFCA degradation kinetics and defluorination. Strong adsorption of long-chained PFCA on porous materials like GAC may render the compound unavailable for reaction with species such as e_aq_^–^. This leads to a process whose reaction rate is limited by the slow desorption rate from the sorbent into the solution. Fine particles (e.g., CNT) and the presence of chromophores (e.g., lignin) attenuate incoming light, thus reducing the photoreactant’s ability to generate reactive species. Consistent with previous studies, light shielding was a strong indicator for reduced PFCA degradation due to lower e_aq_^–^ generation.^34,42^ Additionally, structurally diverse materials like lignin could induce transformation of the e_aq_^–^ source chemical during photochemical processes, which may further reduce e_aq_^–^ generation rates. Finally, materials with electron-withdrawing groups (e.g., lignin) could act as potent scavengers, thus competing for e_aq_^–^ with target compounds. These processes combine to compromise ARP efficiency and PFCA mineralization by decreasing e_aq_^–^ steady-state concentrations. However, the sorbent provides an essential means to first concentrate PFAS, often present at trace concentrations in large volumes of water that need to be treated.

Results indicate that a regeneration scheme using UV/sulfite would be most effective in the absence of sorbents. The PFCAs were largely desorbed for lignin when the pH and ionic strength were increased for the photochemical ARP treatment. Separating the regenerated lignin sorbent (e.g., by filtration or sedimentation) would allow for rapid and efficient PFAA destruction by hydrated electrons. Tunable sorbents like this could be readily scaled up into water treatment for on-site concentration, regeneration, and destruction of PFAA. Several studies are exploring such technologies, for example, through electrochemical redox polymers that efficiently control the capture and release of long- and short-chained PFAA.^82−84^

A critical finding worth further investigation is that PFCA sorbed on activated carbon or CNTs is unlikely to degrade through chemical means, even with small, highly reactive, and diffusive species like e_aq_^–^. This suggests that the activity and influence of e_aq_^–^ within carbon particles is significantly lower than in the bulk solution. Previous studies on GAC regeneration using chemical reactions claim that target compounds must desorb into the bulk solution before reacting.^55−57^ However, it is still unclear whether the lack of surface reactivity is due to limited penetration and activity of the reactive species in the material or to compound-surface interactions that inhibit the reaction, e.g., preventing the reaction by shielding the most reactive α carbon in PFCAs.^59,60^ This result emphasizes the challenge of managing PFAS-laden solids and of achieving the long-term goal of complete defluorination.^85^

Based on our results, short-chained PFAA are more likely to encounter e_aq_^–^ because of their increased water solubility. Nevertheless, some short-chained PFAA tend to be less reactive and thus more vulnerable to e_aq_^–^ quenching by the sorbent and other natural water constituents.^22,46,75^ Short-chained PFAS are increasingly used as replacements and generated in the environment from precursor transformation, thus impacting the performance of concentration and destruction strategies.^86,87^

We encourage future research into sustainable tunable sorbents that can simultaneously address long- and short-chained PFAS while regaining their sorption capacity after being easily regenerated to use ARP for on-site PFAS mineralization. Research is needed to elucidate the mechanism of reactivity and deactivation of PFAS on surfaces. These results have branching implications for PFAS-laden solids such as soil and in situ contaminant barriers requiring regeneration to avoid pollutant breakthrough.

## Supporting Information Available

Definition of the hydrated electron formation rate, dark controls, extraction efficiencies, additional details on analytical methods, quality assurance and quality control, MCAA adsorption on particles, pictures of suspensions in the photoreactor, fluorine mass balances and detected transformation PFCA products, desorption rate data and analysis, raw degradation kinetic data and pseudo-first-order rate constants for PFOA and PFBA, MCAA pseudo-first-order rate constants as a function of its initial concentration with linear regression parameters and statistics, 254 nm absorbance data and analysis, reported bimolecular rate constants, sample calculations, derivation of the scavenging capacity ratios, PFCA adsorption onto particles before and after UV/sulfite treatment, and detailed results from the kinetic models (PDF)
