# Supplementary material for: Carbon Adsorbent Properties Impact Hydrated Electron Activity and Perfluorocarboxylic Acid (PFCA) Destruction
Source: ACS ES T Eng. 2024 Aug 2;4(9):2220–33. doi: 10.1021/acsestengg.4c00211 (PMC11406532; doi:10.1021/acsestengg.4c00211)
Supplement: Supplementary file 1 — ee4c00211_si_001.pdf [file ee4c00211_si_001.pdf]

Supporting Information

Carbon Adsorbent Properties Impact Hydrated Electron Activity and Perfluorocarboxylic Acids (PFCA) Destruction

Hosea A. Santiago-Cruz[a], Zimo Lou[a,b], Jiang Xu[a,c], Ryan C. Sullivan[d,e], Bailey B. Bowers[d,f], Rachel A. Molé[a], Wan Zhang[g], Jinghao Li[g,h,i], Joshua S. Yuan[h], Susie Y. Dai[g], Gregory V. Lowry[a,*]

[a] *Department of Civil and Environmental Engineering, Carnegie Mellon University, Pittsburgh, Pennsylvania 15213, USA*
[b] *Collaborative Innovation Center of Yangtze River Delta Region Green Pharmaceuticals, Zhejiang University of Technology, Hangzhou 310014, China*
[c] *College of Environmental and Resource Sciences, Zhejiang University, Hangzhou 310058, China*
[d] *Department of Chemistry, Carnegie Mellon University, Pittsburgh, Pennsylvania 15217, USA*
[e] *Department of Mechanical Engineering, Carnegie Mellon University, Pittsburgh, Pennsylvania 15217, USA*
[f] *Department of Chemistry and Biochemistry, Oberlin College, Oberlin, Ohio 44074, USA*
[g] *Department of Plant Pathology and Microbiology, Texas A&M University, College Station, Texas 77843, USA*
[h] *Department of Energy, Environmental, and Chemical Engineering, McKelvey School of Engineering, Washington University in St. Louis, St. Louis, Missouri 63130-4899, USA*
[I] *Department of Chemical and Paper Engineering, Western Michigan University, Kalamazoo, Michigan 49008, United States*

Table of Contents

Text S1. Definition of hydrated electron formation rate ($\boldsymbol{R f e a q-}$)........3

Text S2. PFCA and MCAA Analysis........7

Text S3. Fluoride Mass Balance........8

Text S4. Sample calculation of Scavenging Capacity (k'_s) and Particle Bimolecular rate constant ($k_{particle}$)........22

Text S5. Scavenging Capacity Ratios........25

Text S6. Sample Calculation of Modeling PFOA degradation........27

Figure S1. MCAA and PFBA recovery.........4
Figure S2. Dark control experiments.........5
Figure S3. Extraction percent efficiency.........6

Figure S4. MCAA mass fraction recovered in the aqueous phase. 9
Figure S5. Suspension pictures in photoreactor. 10
Figure S6. Percent fluorine mass balance of PFOA degradation. 11
Figure S7. Detected transformation PFCA products of PFOA reaction with UV/SO_3^{2-} in the presence of (A) GAC, (B) CNT, and (C) Lignin. 12
Figure S8. Desorption rate and reaction modeling 13
Figure S9. Fluoride mass balance for the reaction of lower initial PFOA concentration in CNTs. 14
Figure S10. PFOA decomposition kinetics 15
Figure S11. PFBA decomposition kinetics. 16
Figure S12. Inverse MCAA k_{obs} as a function of initial MCAA concentration. 18
Figure S13. PFOA kinetics of filtered water after particle exposure to UV/sulfite 24
Figure S14. Adsorption of PFOA and PFBA onto particles before and after UV/sulfite exposure 26
Figure S15. PFOA kinetic modeling with bimolecular rate constant taken from the most recent literature values. 29

Table S1. Optimized MS/MS transitions and Method Detection Limits (MDL) 7
Table S2. Measured pseudo-first-order rate constants for PFOA and PFBA for all particle systems. Standard error (SE) was obtained from the linear regression of the replicate experiments. 17
Table S3. Linear regression parameters and statistics of Figure S12. 19
Table S4. Estimated mean hydrated electron formation rates. 19
Table S5. Total absorbance at 254nm of sulfite (20mM) solutions at pH 10 at varying particle (CNT and Lignin) concentrations. Fraction of sulfite absorbance (**A_{so3}/A_T**) and fraction of total light absorbance by sulfite. 20
Table S6. Percentage of total light absorbed by sulfite (20mM) at pH 10 with increasing particle concentration. 20
Table S7. Literature values for bimolecular rate constants of target compounds used in this study with e^-_{aq} 21
Table S8. Estimated mean hydrated electron formation rates ($Rfeaq -$), scavenging capacity (k'_s) for each system, scavenging capacity ratios for each over the no particle case ($ks, Particle'ks, No\ Particles'$), and weight normalized particle bimolecular rate constant with hydrated electrons ($k_{particle}$) with standard errors (SE). 23
Table S9. Measured pseudo-first-order rate constants (k_{obs}) for PFOA degradation in filtered water after particle exposure to UV/sulfite and the estimated scavenging capacity ratios for each over the no particle case ($ks, Particle'ks, No\ Particles'$). Standard error (SE) for k_{obs} was obtained from the linear regression of the replicate experiments. 24
Table S10. Values for $Rfeaq -$ and k'_s used in the model. 27
Table S11. **Fastest** PFOA kinetic model calculated parameters using $Rfeaq -$ maximum and k'_S minimum for each condition, values displayed in Table S8. 30
Table S12. Slowest PFOA kinetic model calculated parameters using $Rfeaq -$ minimum and k'_S maximum for each condition, values displayed in Table S8. 31

Text S1. Definition of hydrated electron formation rate ($R_f^{e_{aq}^-}$)

Hydrated electron formation rate ($R_f^{e_{aq}^-}$) under monochromatic light sensitization are described by the following relationship:

$$R_f^{e_{aq}^-} = \frac{\emptyset I_o}{V}(1 - 10^{-A_T})\left(\frac{A_{SO_3^{2-}}}{A_T}\right)$$

Where $A_{SO_3^{2-}}$ is the absorbance of light (254nm) by sulfite (SO_3^{2-}) at pH10, A_T is the total absorbance of the solution, I_o is the photon flux (E/s), $\emptyset$ is the quantum yield of sulfite to generate e^-_{aq} (0.116 mol/E),[1] and V is the volume of the solution. Through the Beer-Lambert relationship, $A_{SO_3^{2-}}$ is linearly related to the light pathlength (l), the molar absorption coefficient at 254nm of sulfite ($\varepsilon_{SO_3^{2-}}$, 18.14 $M^{-1}cm^{-1}$),[1] and the concentration of sulfite ($C_{SO_3^{2-}}$):

$$A_{SO_3^{2-}} = l\ \varepsilon_{SO_3^{2-}}\ C_{SO_3^{2-}}$$

The total absorbance of the solution, A_T, is the sum of the absorbances of all components in the mixture in the given wavelength of light, 254nm. Here, we'll define it as $A_{SO_3^{2-}}$ plus the absorbance of everything else in solution (a_t) which can include constituents in the water matrix and our particles:

$$A_T = A_{SO_3^{2-}} + a_t$$

The expanded relationship for $R_f^{e_{aq}^-}$ is as follows:

$$R_f^{e_{aq}^-} = \frac{\emptyset I_o}{V}\left(1 - 10^{-\left[\left(l\ \varepsilon_{SO_3^{2-}} C_{SO_3^{2-}}\right)+a_t\right]}\right)\left(\frac{l\ \varepsilon_{SO_3^{2-}} C_{SO_3^{2-}}}{\left(l\ \varepsilon_{SO_3^{2-}} C_{SO_3^{2-}}\right) + a_t}\right)$$

For the purpose of our analysis, we define the fraction of light absorbed by the sulfite sensitizer ($f_{abs,SO_3^{2-}}$). We can simplify the $R_f^{e_{aq}^-}$ equation by including $f_{abs,SO_3^{2-}}$ as follows:

$$R_f^{e_{aq}^-} = \frac{\emptyset I_o}{V} f_{abs,SO_3^{2-}}$$

To estimate the percent change in $R_f^{e_{aq}^-}$ based on the impact of $f_{abs,SO_3^{2-}}$ with the introduction of particles, we performed the following calculation assuming that $\frac{\emptyset I_o}{V}$ remains constant despite the addition of particles:

$$Percent\ Change = 100 * \left(1 - \frac{R_{f,Particles}^{e_{aq}^-}}{R_{f,No\ particles}^{e_{aq}^-}}\right) = 100 * \left(1 - \frac{f_{abs,SO_3^{2-},Particles}}{f_{abs,SO_3^{2-},No\ Particles}}\right)$$

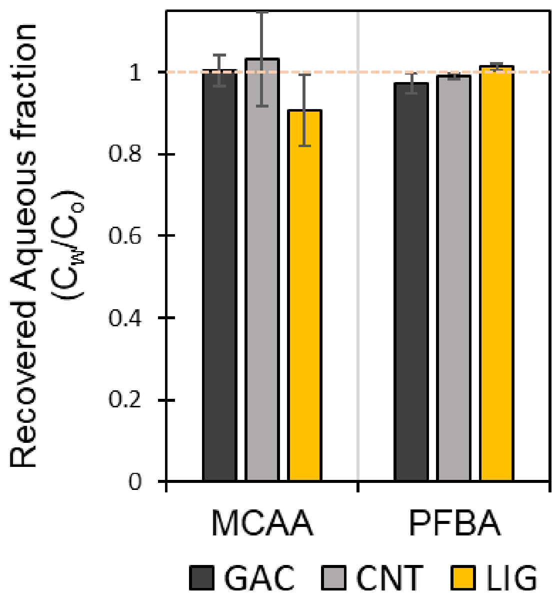

Figure S1. MCAA and PFBA recovery.
MCAA (C_o=10 ppm) and PFBA (C_o=12uM) mass fraction recovered in the aqueous phase after 24 hours in suspensions of CNTs, GAC, or Lignin in 20mM sulfite (SO_3^{2-}) solution at pH 10. The dashed reference line indicates 100% mass fraction recovered in water.

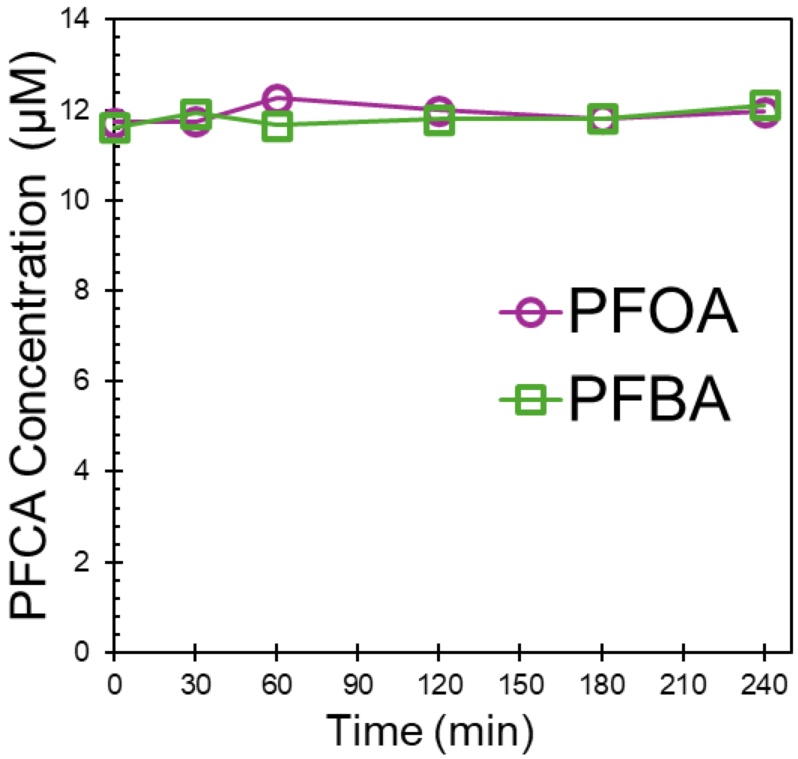

Figure S2. Dark control experiments.
Dark control experiments of PFOA and PFBA with initial concentrations of 12µM, 20 mM sulfite, and pH10 without particles. The solutions were constantly stirred (800rpm) in the glass photoreactor throughout the experiment. Markers represent single measured aqueous samples at each time point.

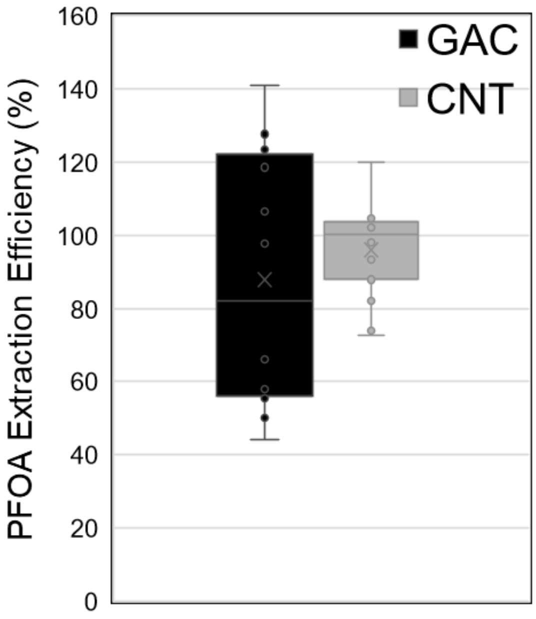

Figure S3. Extraction percent efficiency.
Percent efficiency of the extraction protocol for PFOA equilibrated in a 1g/L suspension of GAC or carbon nanotubes (CNT) in 20mM SO_3^{2-} solution at pH 10. The X marker represents the mean, and the circle markers represent individual samples.

$$Extraction\ Efficiency\ (\%) = 100 * \left(\frac{C_s^{Exp}}{C_s^{Theo}}\right)$$

$$C_s^{Theo} = C_{aq}^{Tot} - C_{aq}^{eq}$$

- C_s^{Exp}: Experimental solid phase concentration measured after the extraction procedure.
- C_s^{Theo}: Theoretical solid phase concentration based on the initial concentration before sorption (C_{aq}^{Tot}) and the equilibrated aqueous phase concentration after sorption (C_{aq}^{eq}).

Text S2. PFCA and MCAA Analysis

Analytical Methods.

To separate background PFAS present in the HPLC pumps from sample peaks, a C_{18} delay column (ZORBAX RR Eclipse Plus, 4.6 x 50 mm, 95 Å, 3.5 µm, Agilent Technologies, US) was installed between the binary pumps and autosampler. A C_{18} analytical column (Poroshell 120 EC-C18, 3.0 x 50 mm, 2.7 µm, Agilent Technologies, US) was used for analyte separation with the column temperature maintained at 40 ^{o}C. Injection volumes were 20 µL. Mobile phases were A: 20 mM ammonium acetate in Milli-Q water and B: MeOH with a flow rate of 0.4 mL/min. Mixtures of PFCA were separated using gradient elution. PFCA were detected with multiple reaction monitoring using quantitative and qualitative ion transitions (Table S3) and quantified via external calibration.

MCAA samples were separated through isocratic elution. Mobile phase ratio of A and B was 98:2 with a flow rate of 0.6 mL/min. MCAA was separated used a larger C_{18} analytical column (Eclipse XDB-C18, 4.6 x 150 mm, 5 µm, Agilent Technologies, US). MCAA was detected using single ion mode and quantified via external calibration. All other conditions were the same as the PFCA method.

Quality Assurance and Quality Control (QA/QC).

Laboratory reagent blanks and controls were run along with continuing calibration verifications (CCVs) at least every 10 samples to ensure calibration validity throughout sample runs. Method detection limits (MDL) were determined using the EPA MDL Procedure, Revision 2. In brief, 7 spiked samples three times the estimated MDL were prepared along with 7 method blank samples. Method blanks returned no numerical results, so the MDL was calculated as the standard deviation of the 7 prepared samples multiplied by t(6,0.99) = 3.143.

Table S1. Optimized MS/MS transitions and Method Detection Limits (MDL)

PFAS	MRM Transitions (*m/z*)		MS Voltages		MDL (µM)
	Precursor ion	Product ion(s)	Fragmentor	Collision Energy	
**PFOA**	413	368.9*, 169	92	0, 12	0.0040
**PFHpA**	363	319*, 169	72	0, 12	0.00089
**PFHxA**	313	269.1*, 118.9	56	0, 12	0.0036
**PFPeA**	263	218.9	56	0	0.012
**PFBA**	213	168.9	56	4	0.0079
**MCAA**	93	35	0	-	1.6

*Indicates quantifier ion if more than one product ion listed

Text S3. Fluoride Mass Balance

The moles of fluorine atoms per PFCA molecule is described as follows: $C_{F,PFCA} = C_{PFCA} * n_F$ Where the fluorine equivalent concentration for each PFCA is $C_{F,PFCA}$, C_{PFCA} is the molar PFCA concentration, and n_F is the number of fluorine atoms in each PFCA. For PFCAs the relationship of fluorine per carbon is as follows:

$$n_F = [2(n_{Carbon} - 1)] + 1$$

Total initial fluorine ($C^o_{F,Total}$)is the total fluorine mass balance **before** particles were added:

$$C^o_{F,Total} = \left(\sum C_{F,PFCA}\right) + \left(C_{F,Free}\right)$$

Where $C_{F,Free}$ is the concentration of free fluoride detected with ISE. All values are normalized to this initial concentration thus providing the maximum value for the fluorine mass balance. The percent PFCA fluorine in a sample at each time t is:

$$\%F^t_{PFCA} = \frac{C^t_{F,PFCA}}{C^o_{F,Total}} \; x \; 100$$

The total fluorine at each time t is:

$$\%F^t_{Total} = \left(\sum \%F^t_{PFCA}\right) + \left(100 \; x \; \frac{C_{F,Free}}{C^o_{F,Total}}\right)$$

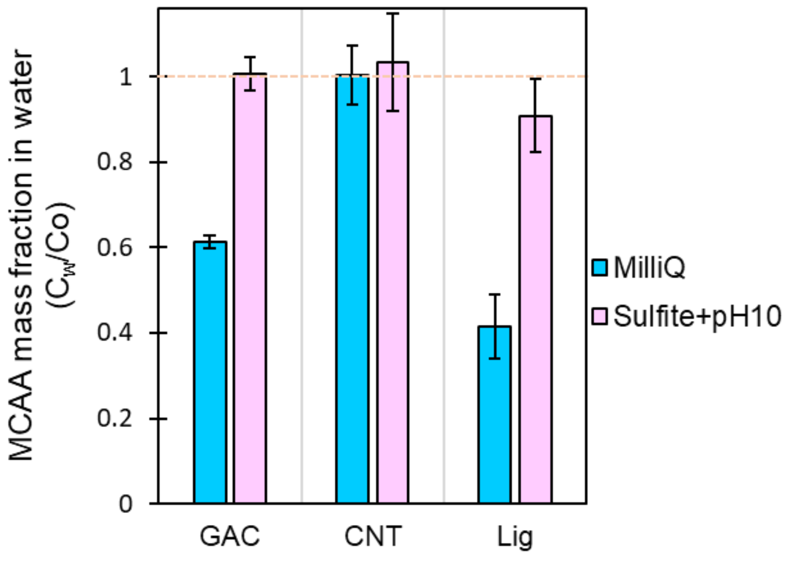

Figure S4. MCAA mass fraction recovered in the aqueous phase.
MCAA (10 ppm) mass fraction recovered in the aqueous phase after 24 hours in suspensions of CNTs, GAC, or Lignin in Milli-Q water at pH 5 or 20mM SO_3^{2-} solution at pH 10 (reaction conditions). Dashed reference line indicates 100% mass fraction recovered in water.

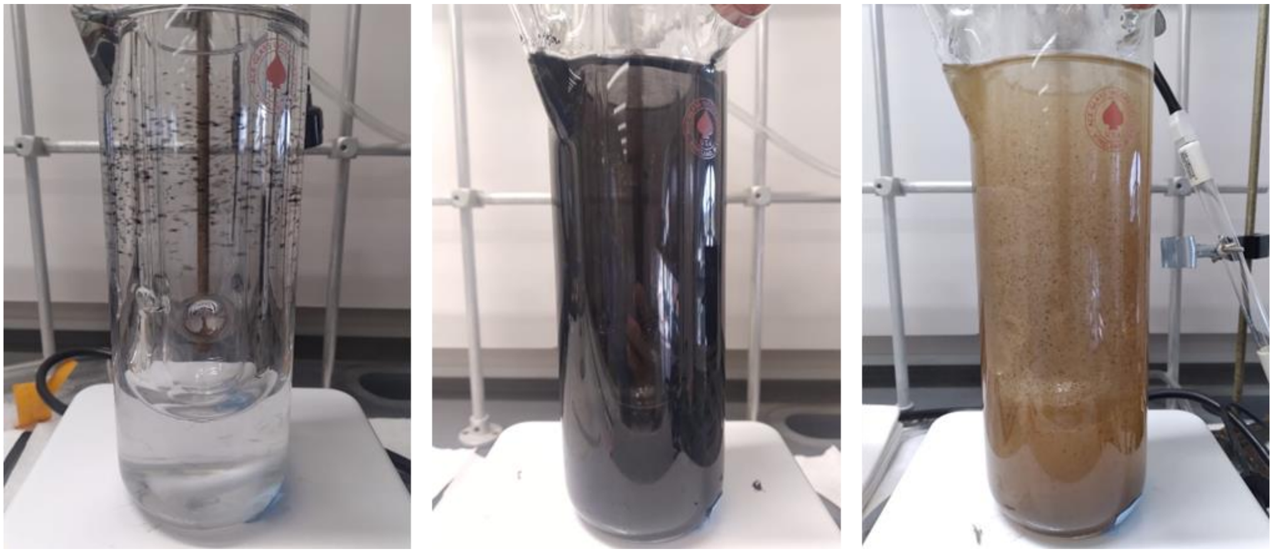

Figure S5. Suspension pictures in photoreactor.
(left to right) Pictures 1 g/L of GAC, CNT, and Lignin suspensions in the photoreactor, respectively.

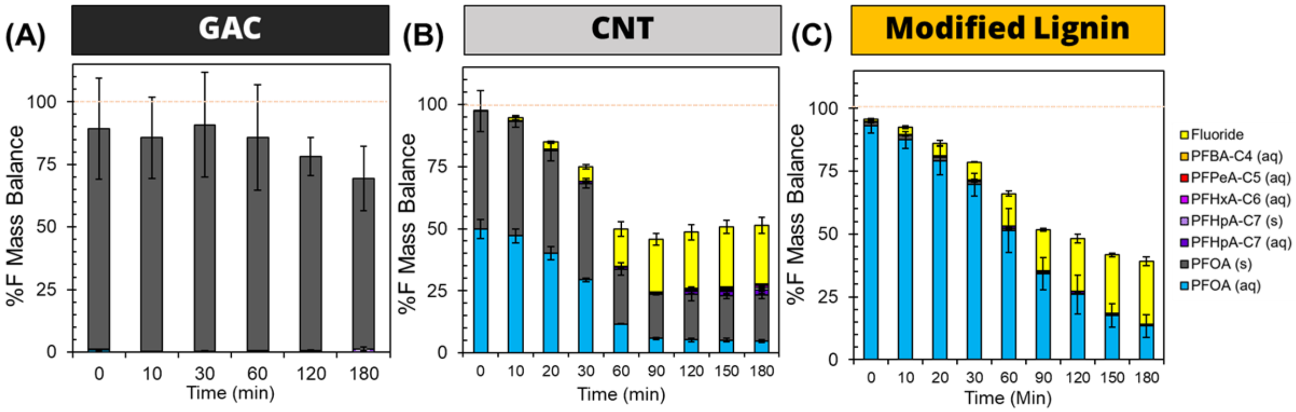

Figure S6. Percent fluorine mass balance of PFOA degradation.
Percent fluorine mass balance of all detected compounds (PFCA n_{carbon}=8-4, and fluoride) during the reaction of 12µM PFOA with 20mM sulfite irradiated with 254nm UV light at pH10 in the presence of 1g/L carbon sorbents (A) GAC, (B) CNT, or (C) Modified Lignin. Note that each PFCA was detected either in the aqueous phase (aq) or extracted from the sorbent (s). The yellow columns illustrate the released fluoride detected. For reference, the orange dashed line in the graph indicates 100% fluoride mass balance determined from the initial fluoride equivalence concentration before particles were introduced. Error bars represent the standard error of replicate measurements (n=2 for all and n=4 for sorbent phase $PFOA_{(s)}$ in GAC). The relatively larger error bars of $PFOA_{(s)}$ in GAC are associated with the error in measuring small particle masses (20-3mg).

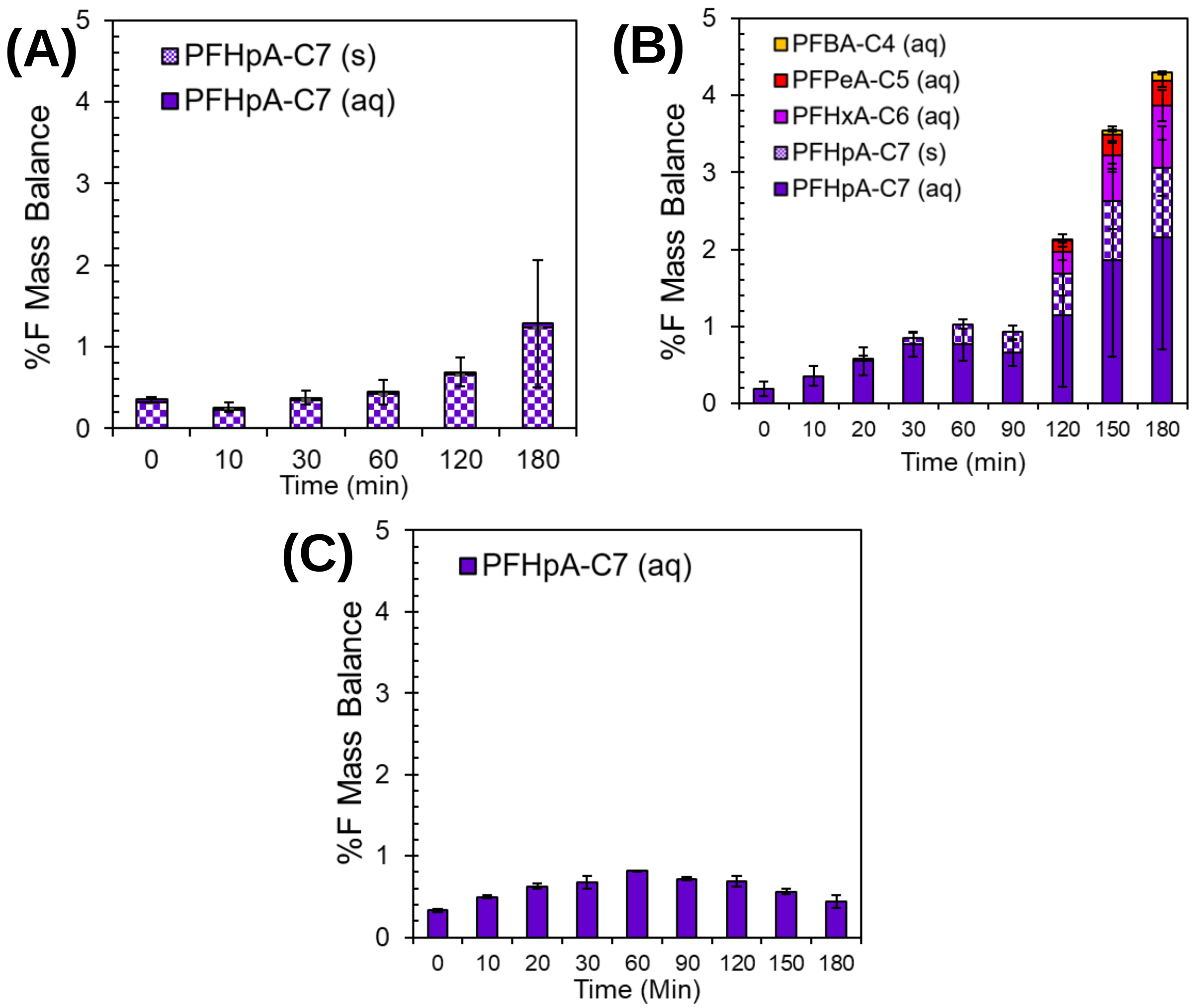

Figure S7. Detected transformation PFCA products of PFOA reaction with UV/SO_3^{2-} in the presence of (A) GAC, (B) CNT, and (C) Lignin.

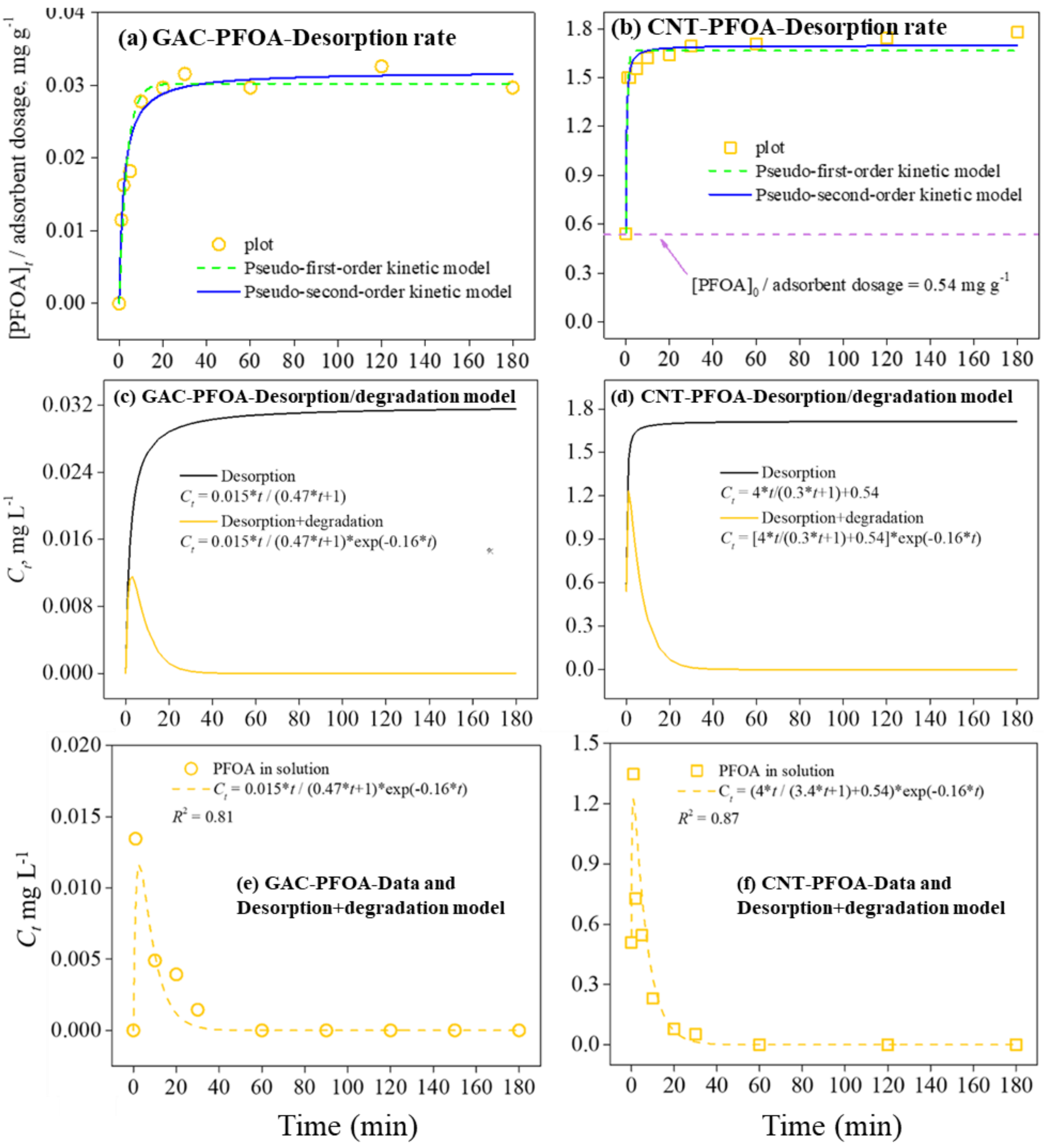

Figure S8. Desorption rate and reaction modeling

(a,b) Fitted desorption rate from 1g/L particles (GAC or CNT) in dark control, (c, d) desorption and reaction modeling, and (e,f) PFOA aqueous concentration data (markers) with modeling profile (dashed lines) for aqueous phase with GAC or CNT. The model was created by measuring the desorption rate of sorbed PFOA (12µM) once the pH was raised to 10 and 20mM SO_3^{-2} was added (a,b). The model was formed by including the desorption and reaction rates (c,d), both parameters measured separately. Data points are degradation experiments with the sorbents (e,f). The model was not fitted to the reaction datapoints shown.

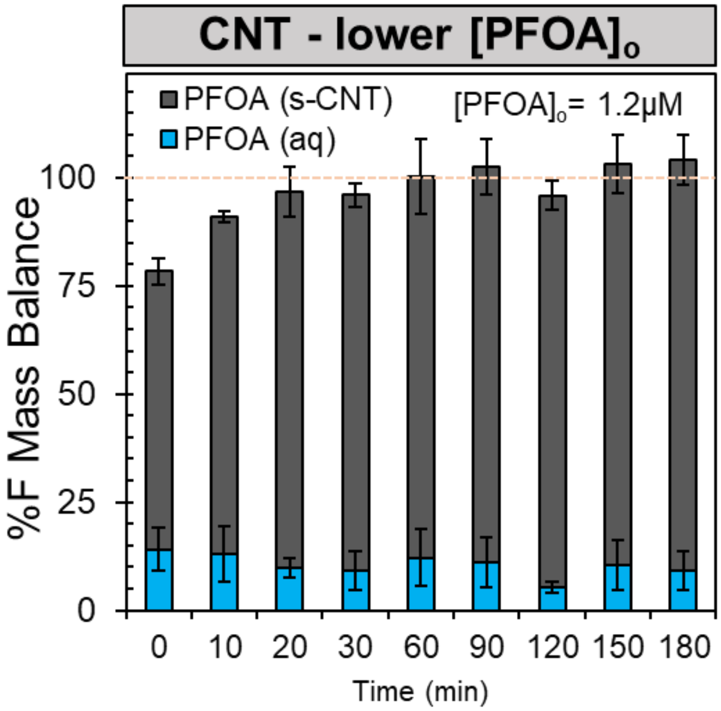

Figure S9. Fluoride mass balance for the reaction of lower initial PFOA concentration in CNTs.
Fluoride mass balance for the reaction of lower initial PFOA concentration (1.2 µM) in UV/SO_3^{2-} treatment in the presence of 1g/L CNT. PFOA was the only compound detected either in the aqueous phase (aq) or extracted from the sorbent (s-CNT). Error bars represent the standard error of replicate measurements. The test was run in 20 mM SO_3^{-2} irradiated with 254 nm UV light at pH 10. For reference, the orange dashed line indicates 100% fluoride mass balance determined from the initial fluoride equivalence concentration of PFOA before particles were introduced.

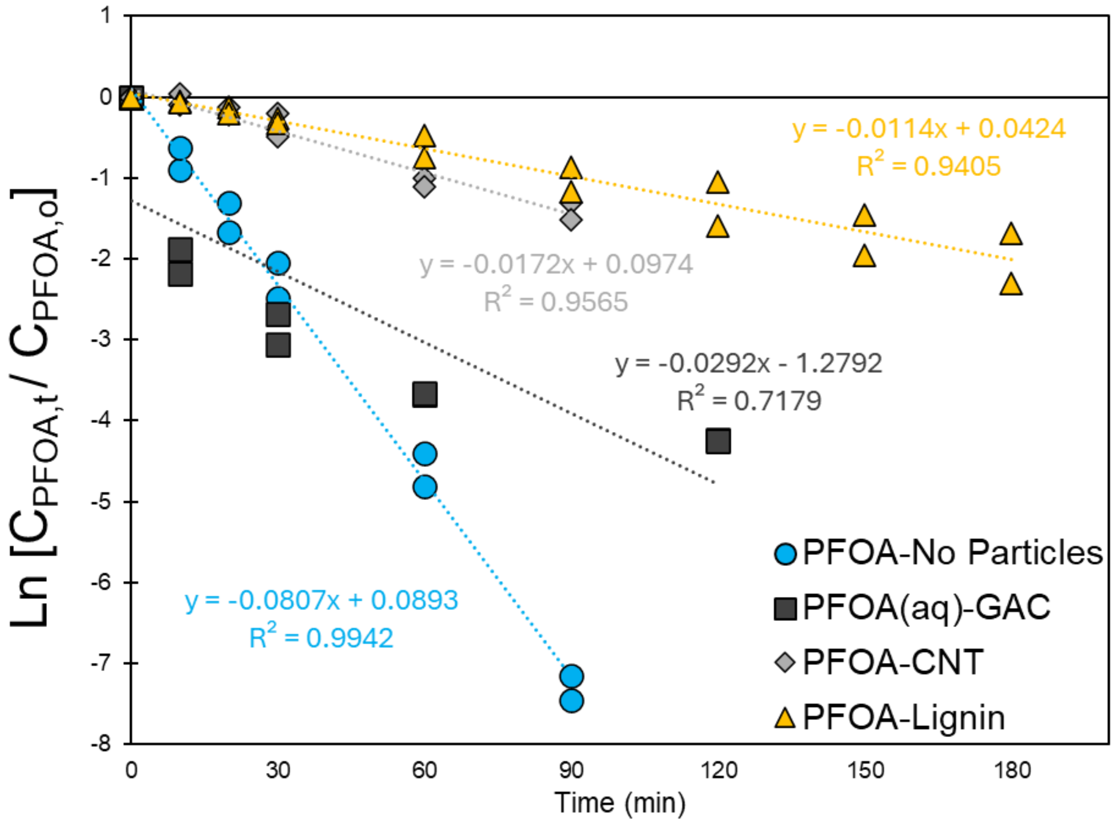

Figure S10. PFOA decomposition kinetics

PFOA ($C_{PFOA,o}$=12 µM) decomposition profile without and with particles (1g/L) under UV/sulfite treatment (20 mM SO_3^{-2} at pH10 irradiated with 254 nm UV). For GAC, measured PFOA concentrations are only from the aqueous phase ($[PFOA]_{0,aq}$=0.1 µM), thus excluding adsorbed PFOA, the rest of the systems (No Particles, CNT, Lignin) is total PFOA concentration (aqueous+sorbed). Markers represent single PFOA measurements. The experiment was run twice; therefore, there are two markers for every time point. The dotted lines are the linear regression of the profile representing pseudo-first-order kinetics for each condition.

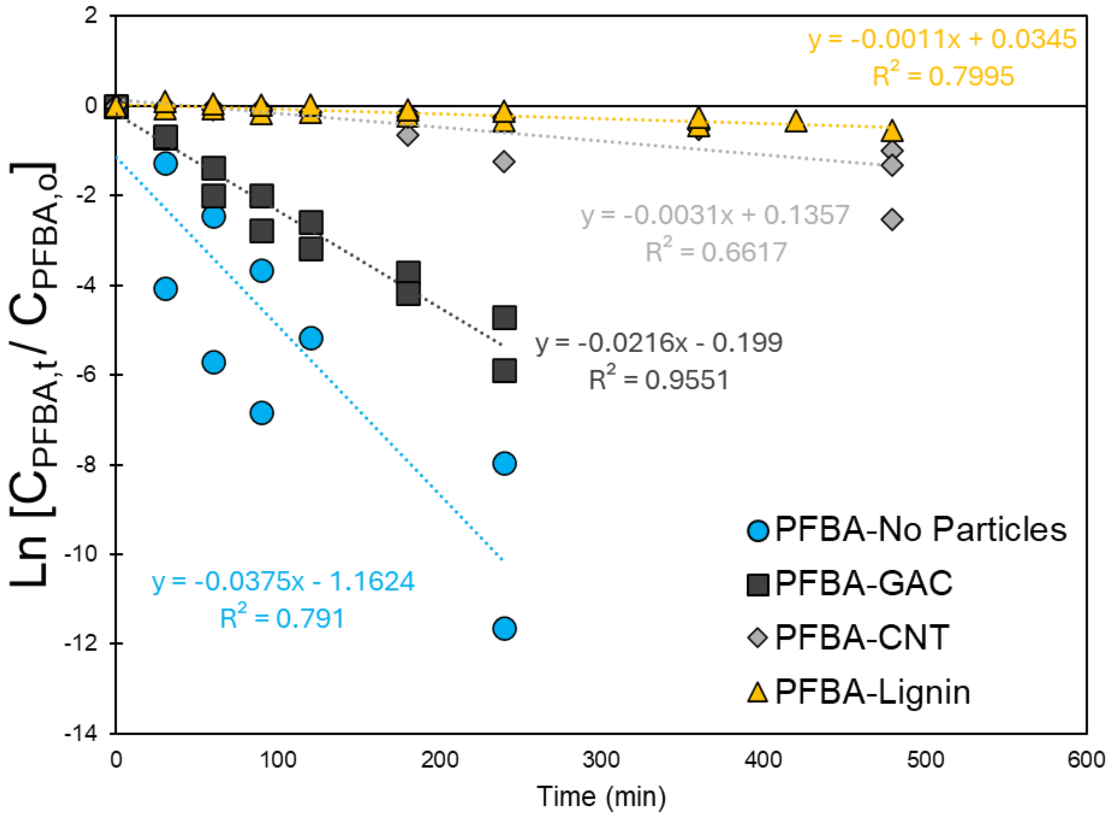

Figure S11. PFBA decomposition kinetics.
PFBA ($C_{PFBA,o}$=12 µM) decomposition profile without and with particles (1g/L) under UV/sulfite treatment (20 mM SO_3^{-2} at pH10 irradiated with 254 nm UV). Because most PFBA is desorbed, aqueous phase concentrations were monitored. Markers represent single PFOA measurements.The experiment was run in replicates (n=2 for No particles, GAC and Lignin: n=2; CNT: n=3); therefore, there are n number of markers for every time point. The dotted line is the linear regression of the profile representing pseudo-first-order kinetics.

Table S2. Measured pseudo-first-order rate constants for PFOA and PFBA for all particle systems. Standard error (SE) was obtained from the linear regression of the replicate experiments.

	k_{obs} ± SE (min^{-1}) PFOA	k_{obs} ± SE (min^{-1}) PFBA
**No Particles**	0.0807 ± 0.0020	0.0375 ± 0.0064
**GAC**	0.0292 ± 0.0065	0.0216 ± 0.0013
**CNT**	0.017 ± 0.001	0.0031 ± 0.0005
**Lignin**	0.0114 ± 0.0007	0.0011 ± 0.0001

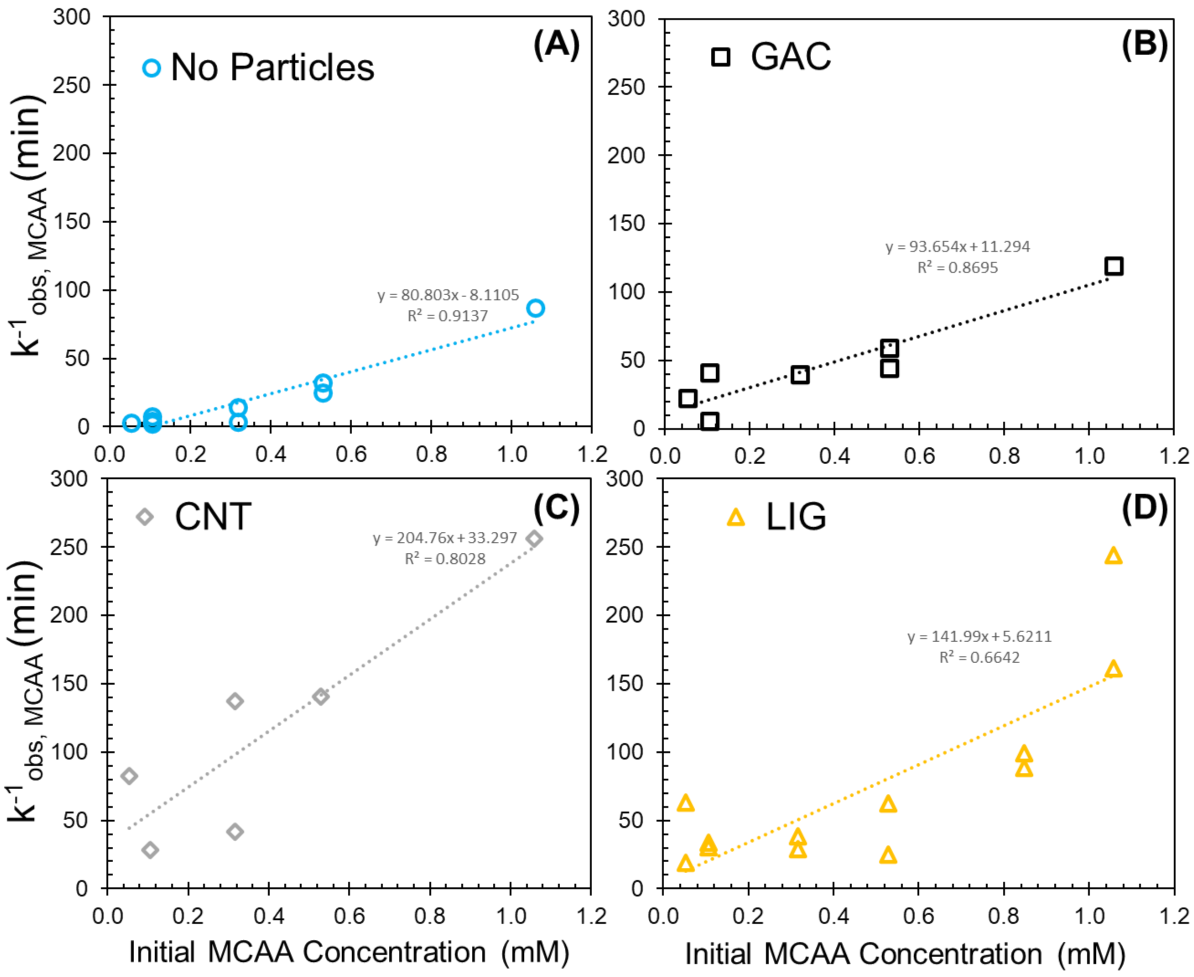

Figure S12. Inverse MCAA k_{obs} as a function of initial MCAA concentration.
Inverse MCAA pseudo-first-order rate constant ($k^{-1}_{obs,\ MCAA}$, min) as a function of initial MCAA concentration. Each marker represents the inverse profile slope of a single degradation experiment where MCAA is decomposed with 20 mM SO_3^{-2} and 254 nm UV light at pH 10 without and with 1 g/L particles. Dashed lines represent the linear regression of the markers. The slope of the linear regression is equivalent to the inverse hydrated electron generation rate ($1/\boldsymbol{R_f^{e^-_{aq}}}$).

Table S3. Linear regression parameters and statistics of Figure S12.

	Slope (min/M)	Standard Error (min/M)	p-value	y-int (min)	Standard Error (min)	p-value	R^2	n
No Particles	8.08×10^4	9.39×10^3	0.000057	-8.1	4.3	0.10	0.91	9
GAC	9.37×10^4	1.62×10^4	0.0022	11.3	8.2	0.23	0.87	7
CNT	2.05×10^5	5.07×10^4	0.0157	33.3	26.3	0.27	0.70	6
LIG	1.51×10^5	4.22×10^4	0.0116	9.925	27.3	0.73	0.68	12

Table S4. Estimated mean hydrated electron formation rates.

	$\mathbf{R_f^{e_{aq}^-}}$ (M/min)	Standard Error (M/min)
No Particles	1.24×10^{-5}	0.144×10^{-5}
GAC	1.07×10^{-5}	0.185×10^{-5}
CNT	0.488×10^{-5}	0.121×10^{-5}
LIG	0.662×10^{-5}	0.185×10^{-5}

Table S5. Total absorbance at 254nm of sulfite (20mM) solutions at pH 10 at varying particle (CNT and Lignin) concentrations. Fraction of sulfite absorbance (**A_{so3}/A_T**) and fraction of total light absorbance by sulfite.

	CNT			Lignin		
**Particle Conc.**	**Total Abs in SO_3^{2-} (A_T)**	**A_{so3}/A_T**	**Fraction light abs by SO_3^{2-}**	**Total Abs in SO_3^{2-} (A_T)**	**A_{so3}/A_T**	**Fraction light abs by SO_3^{2-}**
**0 g/l**	0.288 (A_{so3})	1	0.485	0.288 (A_{so3})	1	0.485
**0.1 g/L**	0.332	0.868	0.464	0.428	0.672	0.422
**1 g/L**	0.609	0.473	0.356	0.944	0.305	0.270

Table S6. Percentage of total light absorbed by sulfite (20mM) at pH 10 with increasing particle concentration.

	Percent Light Abs by SO_3^{2-} (%)	
**Particle Conc.**	**CNT**	**Lignin**
0 g/l	48.5(±2.0)	48.5(±2.0)
0.1 g/L	46.4(±1.0)	42.2(±1.0)
1 g/L	35.6(±2.0)	27.0(±1.0)

Table S7. Literature values for bimolecular rate constants of target compounds used in this study with e^-_{aq}

Compound	k ($M^{-1}s^{-1}$)	Reference
**PFOA**	5.10 x 10^7	Huang, L.; Dong, W.; Hou, H. Investigation of the Reactivity of Hydrated Electron toward Perfluorinated Carboxylates by Laser Flash Photolysis. *Chem Phys Lett* **2007**, *436* (1–3)
	1.70 x 10^7	Szajdzinska-Pietek, E.; Gebicki, J. L. Pulse Radiolytic Investigation of Perfluorinated Surfactants in Aqueous Solutions. *Research on Chemical Intermediates* **2000**, *26*, 897–912
	7.10 x 10^8	Maza, W. A.; Breslin, V. M.; Owrutsky, J. C.; Pate, B. B.; Epshteyn, A. Nanosecond Transient Absorption of Hydrated Electrons and Reduction of Linear Perfluoroalkyl Acids and Sulfonates. *Environ Sci Technol Lett* **2021**, *8* (7), 525–530
**PFBA**	1.27 x 10^7 (pH 9.2)	C.K. Amador et al. Chemosphere 311 (2023) 136918
	1.29 x 10^7 (pH 12)	C.K. Amador et al. Chemosphere 311 (2023) 136918
**MCAA**	1.00 x 10^9	Journal of Physical and Chemical Reference Data 17, 513–886 (1988). https://doi.org/10.1063/1.555805

Text S4. Sample calculation of Scavenging Capacity (k'_s) and Particle Bimolecular rate constant ($k_{particle}$)

Hydrated Electron Steady State Concentration:

$$[e_{aq}^-]_{ss} = \frac{k_{obs,PFBA}}{k_{PFBA}}$$

$$[e_{aq}^-]_{ss,No\ Part} = \frac{0.0375\ (\pm 0.0049) min^{-1} \left(\frac{1\ min}{60\ s}\right)}{1.28\ (\pm 0.04)\ x\ 10^7 M^{-1} s^{-1}}$$

$$[e_{aq}^-]_{ss,No\ Part} = 4.88\ (\pm 0.66)\ x 10^{-11} M$$

Scavenging Capacity:

$$k'_s = \frac{R_f^{e_{aq}^-}}{[e_{aq}^-]_{ss}} - k_{PFBA} C_{PFBA,o}$$

$$k'_{s,\ No\ Part} = \left[\frac{\left(1.24\ (\pm 0.144)\ x 10^{-5} \frac{M}{min}\right)\left(\frac{1\ min}{60\ s}\right)}{4.88\ (\pm 0.66)\ x 10^{-11} M}\right] - (1.28\ (\pm 0.04) x 10^7 M^{-1} s^{-1})(12 x 10^{-6} M)$$

$$k'_{s,No\ Part} = 0.407\ (\pm 0.088)\ x\ 10^4\ s^{-1}$$

Particle Bimolecular Rate Constant:

$$k_{GAC} = \frac{\left(k'_{s,GAC} - k'_{s,No\ Part}\right)}{m_{GAC}} = \frac{(0.619 \pm 0.118 x\ 10^4\ s^{-1} - 0.407 \pm 0.088\ x\ 10^4\ s^{-1})}{1 \frac{g}{L}}$$

$$k_{GAC} = 0.212\ (\pm 0.147) x\ 10^4 \frac{L}{g\ s}$$

Table S8. Estimated mean hydrated electron formation rates ($R_f^{e_{aq}^-}$), scavenging capacity (k'_s) for each system, scavenging capacity ratios for each over the no particle case ($\frac{k'_{s,Particle}}{k'_{s,No\ Particles}}$), and weight normalized particle bimolecular rate constant with hydrated electrons ($k_{particle}$) with standard errors (SE).

.

	$R_f^{e_{aq}^-}$ (10^{-5} M/min)	SE ($\pm 10^{-5}$ M/min)	k'_s (10^4 s^{-1})	SE ($\pm 10^4$ s^{-1})	$\frac{k'_{s,Particle}}{k'_{s,No\ Particle}}$	SE (±)	$k_{particle}$ (10^4 L g^{-1} s^{-1})	SE ($\pm 10^4$ L g^{-1} s^{-1})
**No Particle**	1.24	0.144	0.407	0.088	-	-	-	-
**GAC**	1.07	0.185	0.619	0.118	1.5	0.4	0.212	0.147
**CNT**	0.488	0.121	2.01	0.587	5	2	1.61	0.590
**Lignin**	0.662	0.185	8.49	2.19	21	7	8.08	2.19

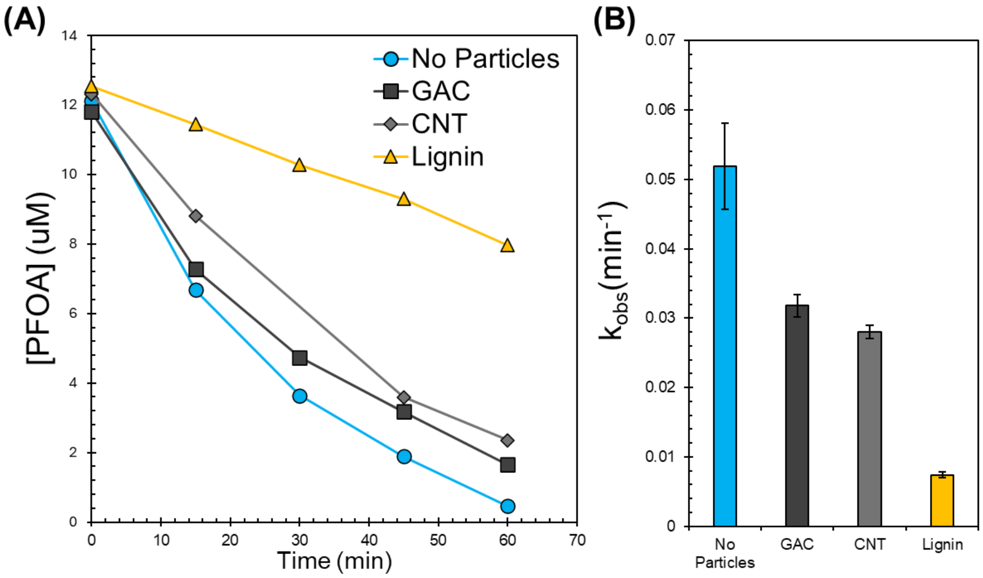

Figure S13. PFOA kinetics of filtered water after particle exposure to UV/sulfite

Spiked PFOA ($C_{PFOA,o}$=12 μM) decomposition profile under UV/sulfite treatment in filtered water from control (no particles) and particle suspensions (1g/L) previously subjected to UV/sulfite (20 mM SO_3^{-2} at pH10 irradiated with 254 nm UV for 3 hours). PFOA spiked filtered water was readjusted to pH10 with H_2SO_4 (1M) as the pH of each system increased after 3 hours of exposure to UV/sulfite. (A) The concentration of PFOA over reaction time and (B) measured pseudo-first-order rate constants (k_{obs}). For (A), each data point represents a single aqueous sampled measurement. For (B), the error bars represent the standard error of each linear regression.

Table S9. Measured pseudo-first-order rate constants (k_{obs}) for PFOA degradation in filtered water after particle exposure to UV/sulfite and the estimated scavenging capacity ratios for each over the no particle case ($\frac{k'_{s,Particle}}{k'_{s,No\ Particles}}$). Standard error (SE) for k_{obs} was obtained from the linear regression of the replicate experiments.

	k_{obs} (min^{-1})	SE (± min^{-1})	$\frac{k_{obs,No\ Particle}}{k_{obs,Particle}} \cong \frac{k'_{s,Particle}}{k'_{s,No\ Particles}}$ (unitless)	SE (±)
**No Particle**	0.052	0.006	-	-
**GAC**	0.032	0.002	1.6	0.2
**CNT**	0.0280	0.0009	1.9	0.2
**Lignin**	0.0074	0.0004	7.0	0.9

Text S5. Scavenging Capacity Ratios

The increase in scavenging capacity of the dissolved species relative to the no particle case can be roughly estimated. To estimate the relative contribution of dissolved species to the total scavenging capacity of each suspension, we calculate the hydrated electron scavenging capacity ratio of the filtered particles suspension exposed to UV/sulfite over the no particle control using the following relationship:

$$\frac{k_{obs,No\ Particle}}{k_{obs,Particle}} = \frac{k_i[e_{aq}^-]_{No\ Particles}}{k_i[e_{aq}^-]_{Particle}} = \frac{\left(\frac{R_{f,No\ Particles}^{e_{aq}^-}}{k_iC_i + k'_{s,No\ Particles}}\right)}{\left(\frac{R_{f,Particle}^{e_{aq}^-}}{k_iC_i + k'_{s,Particle}}\right)}$$

Because all the particles are filtered out of the solution, it is assumed that the hydrated electron generation rate is the same for these samples as the particles are not affecting light penetration. For the filtered solution after particle UV/sulfite exposure:

$$R_{f,No\ Particles}^{e_{aq}^-} \cong R_{f,Particle}^{e_{aq}^-}$$

$$\frac{k_{obs,No\ Particle}}{k_{obs,Particle}} \cong \frac{(k_iC_i + k'_{s,Particle})}{(k_iC_i + k'_{s,No\ Particles})}$$

As the PFCA contribution to hydrated electron consumption is smaller than those of the scavengers ($k_iC_i << k'_S$) the equation can be simplified to the following working equation:

$$\frac{k_{obs,No\ Particle}}{k_{obs,Particle}} \cong \frac{k'_{s,Particle}}{k'_{s,No\ Particles}}$$

This last equation is the relationship used to estimate the ratio for the filtered samples. Calculated values are reported in Table S9.

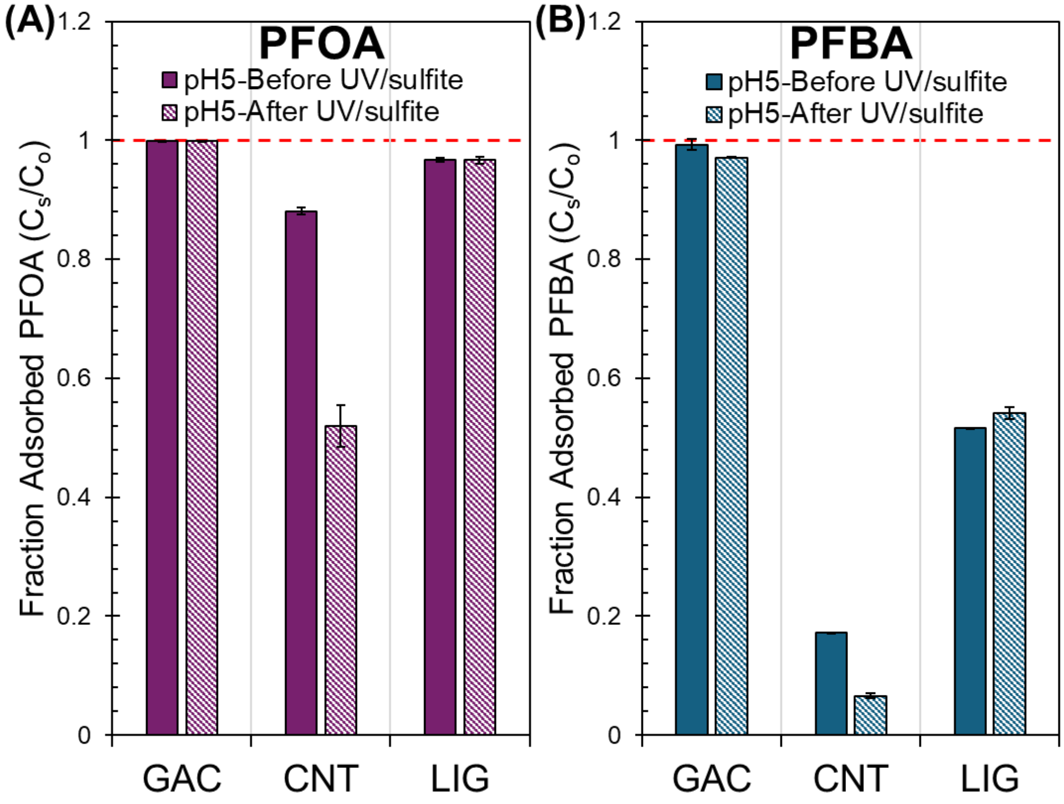

Figure S14. Adsorption of PFOA and PFBA onto particles before and after UV/sulfite exposure

Adsorbed fraction ($C_S/C_o = 1 - C_w/C_o$) of 12 µM (C_o) (A) PFOA and (B) PFBA on 1g/L of GAC, CNT, and lignin (LIG) in solutions of Milli-Q water at pH5. Adsorption was performed on particles before (solid colored bars) or after (stripped colored bars) particles were exposed to UV/sulfite (pH10, 20mM SO_3^{2-}, 245nm light irradiation for 3 hours). Particles exposed to UV/sulfite were filtered from solution, rinsed with Milli-Q water, and then freeze dried before adsorption test. For the adsorption test, the suspension pH was adjusted to 5 with a 1mM hydrochloric acid solution. Aqueous phase samples were measured after 24 hours of adsorption. Error bars represent the standard error of experimental duplicates. Dashed reference lines indicate 100% sorbed mass fraction.

Table S10. Values for $R_f^{e_{aq}^-}$ and k'$_s$ used in the model.

	$R_f^{e_{aq}^-}$ (M/min)			k'$_s$ (min^{-1})		
	**minimum**	**maximum**	**mean**	**minimum**	**maximum**	**mean**
**No Particles**	1.09E-05	1.38E-05	1.24E-05	1.91E+05	2.97E+05	2.44E+05
**GAC**	8.83E-06	1.25E-05	1.07E-05	3.00E+05	4.42E+05	3.71E+05
**CNT**	3.67E-06	6.09E-06	4.88E-06	8.56E+05	1.56E+06	1.21E+06
**LIG**	4.77E-06	8.47E-06	6.62E-06	3.78E+06	6.41E+06	5.09E+06

Maximum = Mean+SE

Minimum = Mean-SE

Text S6. Sample Calculation of Modeling PFOA degradation

No Particles, fastest rates:

- k_{PFOA} = 2.04 x 10^9 M^{-1} min^{-1}
- Max e$^-_{aq}$ Formation: $R_{f,No\ Part}^{e_{aq}^-}$= 1.38 x 10^{-5} M/min
- Minimum scavenging: k'$_{s,\text{ No Part}}$ = 1.91 x 10^5 min^{-1}

Initial Conditions t=0 min:

Total Consumption Rate

$$k'_{C,t} = k_i C_{i,t} + k'_s$$

$$k'_{C,No\ Part,t=0} = [(2.04x10^9\ M^{-1}min^{-1})(12.0x10^{-6}\ M)] + 1.91x10^5 min^{-1}$$

$$k'_{C,No\ Part,t=0} = 2.15x10^5\ min^{-1}$$

Hydrated Electron Concentration

$$[e_{aq}^-]_t = \frac{R_f^{e_{aq}^-}}{k'_{C,t}}$$

$$[e_{aq}^-]_{No\ Part,t=0} = \frac{1.38x10^{-5}\ \frac{M}{min}}{1.91x10^5\ min^{-1}}$$

$$[e_{aq}^-]_{No\ Part,t=0} = 7.23x10^{-11}\ M$$

Observed Rate Constant

$$k_{obs,t} = k_{PFOA}[e_{aq}^{-}]_t$$

$$k_{obs,No\ Part,t=0} = (2.04x10^{9}\ M^{-1}min^{-1})(7.50x10^{-11}\ M)$$

$$k_{obs,No\ Part,t=0} = 0.147\ min^{-1}$$

Rate Expression

$$C_{i,t} = C_{i,o}exp\ [-k_{obs,t} * t]$$

$$C_{PFOA,t=1} = (12.0x10^{-6}\ M)\ exp\ [-(0.147\ min^{-1}) * (1\ min)]$$

$$C_{PFOA,t=1} = 10.4x10^{-6}\ M$$

t = 5 min

Total Consumption Rate

$$k'_{C,t} = k_i C_{i,t_{previous}} + k'_s$$

$$k'_{C,No\ Part,t=5} = k_{PFOA}C_{PFOA,t=1} + k'_{s,No\ Part}$$

$$k'_{C,No\ Part,t=5} = [(2.04x10^{9}\ M^{-1}min^{-1})(10.4x10^{-6}\ M)] + 1.91x10^{5}min^{-1}$$

$$k'_{C,No\ Part,t=5} = 2.12x10^{5}\ min^{-1}$$

Hydrated Electron Concentration

$$[e_{aq}^{-}]_{No\ Part,t=5} = \frac{1.38x10^{-5}\frac{M}{min}}{2.12x10^{5}\ min^{-1}}$$

$$[e_{aq}^{-}]_{No\ Part,t=5} = 6.51x10^{-11}\ M$$

Observed Rate Constant

$$k_{obs,No\ Part,t=5} = (2.04x10^{9}\ M^{-1}min^{-1})(6.51x10^{-11}\ M)$$

$$k_{obs,No\ Part,t=5} = 0.133\ min^{-1}$$

Rate Expression

$$C_{i,t} = C_{i,t_{previous}}exp\ [-k_{obs,t} * (t - t_{previous})]$$

$$C_{PFOA,t=5} = (10.4x10^{-6}\ M)\ exp\ [-(0.133\ min^{-1}) * (5\ min - 1\ min)]$$

$$C_{PFOA,t=5} = 6.09x10^{-6}\ M$$

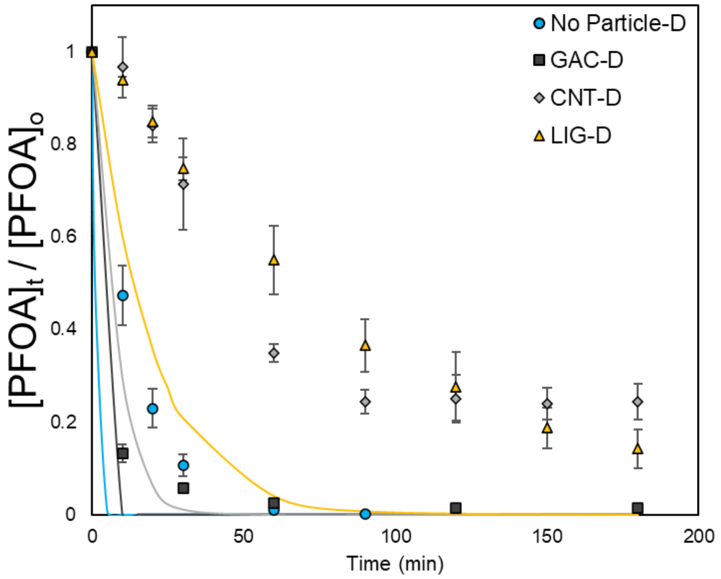

Figure S15. PFOA kinetic modeling with bimolecular rate constant taken from the most recent literature values.

Modeling PFOA transformation rates in UV/SO_3^{-2} with estimated values of $R_f^{e_{aq}^-}$ and $k'_{S,0}$. Measured PFOA degradation is illustrated by the symbols while the solid lines represent the modeled degradation for each system (No particles, GAC, CNT, and Lig). Experiments were run at pH 10 with 20mM SO_3^{-2} and $[PFOA]_0$=12 µM, irradiated with 254nm light at ambient temperature (20°C). Bimolecular rate constant k_{PFOA}= 7.10 x 10^8 $M^{-1}s^{-1}$ taken from the most recent literature values (Maza etal, 2021). Markers are the means of replicate measurements and error bars represent their standard error. *In the GAC system, measured PFOA concentrations are only from the aqueous phase ($[PFOA]_{0,aq}$=0.1µM) thus excluding adsorbed PFOA.

Table S11. **Fastest** PFOA kinetic model calculated parameters using $R_f^{e_{aq}^-}$ maximum and k'_S minimum for each condition, values displayed in Table S8.

Particle	T (min)	Consumption Rate, $k'_{C,t}$ (min^{-1})	$[e^-_{aq}]_t$ (M)	$k_{obs,t}$ (min^{-1})	C_t (M)	$C_t/C_{t=0}$
No Particles	0	2.16E+05	6.40E-11	1.31E-01	1.20E-05	1.00E+00
	1	2.16E+05	6.40E-11	1.31E-01	1.05E-05	8.78E-01
	5	2.13E+05	6.49E-11	1.32E-01	6.20E-06	5.17E-01
	10	2.04E+05	6.77E-11	1.38E-01	3.11E-06	2.59E-01
	20	1.98E+05	6.99E-11	1.43E-01	7.48E-07	6.23E-02
	30	1.93E+05	7.16E-11	1.46E-01	1.73E-07	1.45E-02
	60	1.92E+05	7.20E-11	1.47E-01	2.11E-09	1.76E-04
	90	1.91E+05	7.22E-11	1.47E-01	2.55E-11	2.12E-06
	180	1.91E+05	7.22E-11	1.47E-01	4.48E-17	3.73E-12
GAC	0	3.00E+05	4.17E-11	8.51E-02	1.00E-07	1.00E+00
	10	3.00E+05	4.17E-11	8.51E-02	4.27E-08	4.27E-01
	15	3.00E+05	4.17E-11	8.51E-02	2.79E-08	2.79E-01
	20	3.00E+05	4.17E-11	8.51E-02	1.82E-08	1.82E-01
	25	3.00E+05	4.17E-11	8.51E-02	1.19E-08	1.19E-01
	30	3.00E+05	4.17E-11	8.51E-02	7.78E-09	7.78E-02
	60	3.00E+05	4.17E-11	8.51E-02	6.05E-10	6.05E-03
	90	3.00E+05	4.17E-11	8.51E-02	4.71E-11	4.71E-04
	180	3.00E+05	4.17E-11	8.51E-02	2.21E-14	2.21E-07
CNT	0	8.80E+05	6.92E-12	1.41E-02	1.20E-05	1.00E+00
	10	8.80E+05	6.92E-12	1.41E-02	1.04E-05	8.68E-01
	20	8.77E+05	6.95E-12	1.42E-02	9.04E-06	7.54E-01
	30	8.74E+05	6.97E-12	1.42E-02	7.84E-06	6.54E-01
	60	8.72E+05	6.99E-12	1.43E-02	5.11E-06	4.26E-01
	90	8.66E+05	7.03E-12	1.43E-02	3.33E-06	2.77E-01
	120	8.63E+05	7.06E-12	1.44E-02	2.16E-06	1.80E-01
	150	8.60E+05	7.08E-12	1.44E-02	1.40E-06	1.17E-01
	180	8.59E+05	7.10E-12	1.45E-02	9.06E-07	7.55E-02
Lignin	0	3.80E+06	2.23E-12	4.54E-03	1.20E-05	1.00E+00
	10	3.80E+06	2.23E-12	4.54E-03	1.15E-05	9.56E-01
	20	3.80E+06	2.23E-12	4.55E-03	1.10E-05	9.13E-01
	25	3.80E+06	2.23E-12	4.55E-03	1.07E-05	8.93E-01
	30	3.80E+06	2.23E-12	4.55E-03	1.05E-05	8.73E-01
	60	3.80E+06	2.23E-12	4.55E-03	9.13E-06	7.61E-01
	90	3.80E+06	2.23E-12	4.55E-03	7.97E-06	6.64E-01
	180	3.79E+06	2.23E-12	4.55E-03	5.29E-06	4.41E-01

Table S12. Slowest PFOA kinetic model calculated parameters using $R_f^{e_{aq}^-}$ minimum and k'$_S$ maximum for each condition, values displayed in Table S8.

Particle	T (min)	Consumption Rate, $k'_{C,t}$ (min^{-1})	$[e^-_{aq}]_t$ (M)	k_{obs} (min^{-1})	C_t (M)	$C_t/C_{t=0}$
No Particles	0	3.21E+05	3.40E-11	6.94E-02	1.20E-05	1.00E+00
	1	3.21E+05	3.40E-11	6.94E-02	1.12E-05	9.33E-01
	5	3.20E+05	3.42E-11	6.98E-02	8.47E-06	7.06E-01
	10	3.14E+05	3.48E-11	7.10E-02	5.94E-06	4.95E-01
	20	3.09E+05	3.54E-11	7.22E-02	2.89E-06	2.40E-01
	30	3.03E+05	3.61E-11	7.37E-02	1.38E-06	1.15E-01
	60	3.00E+05	3.65E-11	7.44E-02	1.48E-07	1.23E-02
	90	2.97E+05	3.68E-11	7.51E-02	1.56E-08	1.30E-03
	180	2.97E+05	3.68E-11	7.51E-02	1.80E-11	1.50E-06
GAC	0	4.42E+05	2.00E-11	4.07E-02	1.00E-07	1.00E+00
	10	4.42E+05	2.00E-11	4.07E-02	6.66E-08	6.66E-01
	15	4.42E+05	2.00E-11	4.07E-02	5.43E-08	5.43E-01
	20	4.42E+05	2.00E-11	4.07E-02	4.43E-08	4.43E-01
	25	4.42E+05	2.00E-11	4.07E-02	3.61E-08	3.61E-01
	30	4.42E+05	2.00E-11	4.07E-02	2.95E-08	2.95E-01
	60	4.42E+05	2.00E-11	4.07E-02	8.69E-09	8.69E-02
	90	4.42E+05	2.00E-11	4.07E-02	2.56E-09	2.56E-02
	180	4.42E+05	2.00E-11	4.07E-02	6.55E-11	6.55E-04
CNT	0	1.58E+06	2.32E-12	4.73E-03	1.20E-05	1.00E+00
	10	1.58E+06	2.32E-12	4.73E-03	1.14E-05	9.54E-01
	20	1.58E+06	2.32E-12	4.73E-03	1.09E-05	9.10E-01
	30	1.58E+06	2.32E-12	4.74E-03	1.04E-05	8.68E-01
	60	1.58E+06	2.32E-12	4.74E-03	9.03E-06	7.53E-01
	90	1.58E+06	2.33E-12	4.75E-03	7.83E-06	6.53E-01
	120	1.58E+06	2.33E-12	4.76E-03	6.79E-06	5.66E-01
	150	1.57E+06	2.33E-12	4.76E-03	5.89E-06	4.91E-01
	180	1.57E+06	2.34E-12	4.77E-03	5.10E-06	4.25E-01
Lignin	0	6.43E+06	7.42E-13	1.51E-03	1.20E-05	1.00E+00
	10	6.43E+06	7.42E-13	1.51E-03	1.18E-05	9.85E-01
	20	6.43E+06	7.42E-13	1.51E-03	1.16E-05	9.70E-01
	25	6.43E+06	7.42E-13	1.51E-03	1.16E-05	9.63E-01
	30	6.43E+06	7.42E-13	1.51E-03	1.15E-05	9.56E-01
	60	6.43E+06	7.42E-13	1.51E-03	1.10E-05	9.13E-01
	90	6.43E+06	7.42E-13	1.51E-03	1.05E-05	8.73E-01
	180	6.43E+06	7.43E-13	1.51E-03	9.14E-06	7.61E-01

References:

1. Li, X.; Ma, J.; Liu, G.; Fang,J.; Yue, S.; Guan,Y.; Chen,L.; Liu,X. Efficient Reductive Dechlorination of Monochloroacetic Acid by Sulfite/UVProcess. *Environ.Sci. Technol*.**2012**,46,7342−9.
